# A distinct subgroup with Schwann-like differentiation presents immune resistance and poor prognosis in plantar melanoma

**DOI:** 10.7150/thno.99526

**Published:** 2025-03-03

**Authors:** Jie Tian, Lu Zhang, Le Zhuang, Pingping Lin, Shenxi Zhang, Yicen Yan, Yu Yang, Guohong Zhang, Hang Li, Binbin Lai

**Affiliations:** 1 Department of Dermatology and Venereology, Peking University First Hospital, Beijing, China.; 2 Institute of Medical Technology, Peking University Health Science Center, Beijing, China.; 3 Biomedical Engineering Department, Institute of Advanced Clinical Medicine, Peking University, Beijing, China.; 4 National Clinical Research Center for Skin and Immune Diseases, Beijing, China.; 5 Beijing Key Laboratory of Molecular Diagnosis on Dermatoses, Beijing, China.; 6 NMPA Key Laboratory for Quality Control and Evaluation of Cosmetics, Beijing, China.; 7 Pathology Department, Shantou University Medical College, Guangdong, China.; 8 Department of Dermatology, Central Hospital Affiliated to Shandong First Medical University, Shandong, China.; 9 State Key Laboratory of Molecular Oncology, Peking University International Cancer Institute, Beijing, China.

**Keywords:** *HMGA2*, Plantar melanoma, Schwann cell-like melanoma, Single-cell multiomics sequencing, Tumor heterogeneity

## Abstract

**Background:** Intratumor heterogeneity in plantar melanoma orchestrates transcriptional programs that contribute to resistance to target- and immuno-therapies. However, the evolution and spatial distribution of cellular subgroups, as well as their effects on immune environment and patient prognosis, remain unclear.

**Methods:** We analyzed 218,021 cells from 20 plantar melanoma and 6 normal samples using single-cell RNA sequencing to reveal the evolutionary characteristics and communication patterns of tumor subgroups. Spatial transcriptomics and multiplex immunohistochemistry (mIHC) were used to map the spatial distribution of these subgroups, with mIHC scores further evaluating their correlation with patient prognosis. Single-cell multiomics analysis identified key transcription factors associated with chromatin accessibility. In addition, survival analysis was performed using bulk RNA sequencing data from 68 melanoma patients.

**Results:** We identified a continuum of subgroups originating from stem cells via transitional and Schwann cell-like precursor states, ultimately reaching a Schwann cell-like state. This evolution trajectory was supported by integrative evidence, including assessments of stemness, transitional states, RNA velocity, and transcription factors. The histological distribution of these subgroups was validated by spatial transcriptomics and multiple IHC. Notably, Schwann cell-like subgroup, regulated by transcription factor *HMGA2*, was associated with immune cell dysregulation and a worse prognosis, including increased invasion and lymph node metastasis. Mechanically, inhibition of *HMGA2* expression blocked the transition to Schwann-like melanoma fate.

**Conclusions:** This study reveals the unique evolutionary trajectory of plantar melanoma, showing its differentiation towards a Schwann-like fate regulated by *HMGA2*, leading to a decline in pigment function, enhanced immune tolerance and an increased propensity for lymph node metastasis.

## Introduction

Melanoma represents a significant clinical challenge due to its high mortality and poor prognosis 1-3, particularly plantar melanoma (PM), which has a significantly lower response rate to immunotherapy and an even worse prognosis 4, 5. Current treatment strategies rely primarily on targeted and immunotherapies; however, limitations arise from patient non-response or drug resistance development 6, 7. Notably, the objective response rate of PM to programmed cell death protein 1 inhibitors is only 14%-18% 8-10. Therefore, unraveling resistance mechanisms and identifying novel therapeutic targets have become the main direction of current research in overcoming the limitations of existing treatments.

Recently, Pozniak 11 *et al.* identified a critical breakthrough in understanding resistance to immunotherapy in melanoma. This discovery comes from a subset of mesenchymal-type melanomas characterized by high expression of transcription factor 4 (*TCF4*) and low expression of microphthalmia-associated transcription factor (*MITF*). Experiments have shown that reducing the expression of *TCF4* significantly increases the sensitivity of melanoma cells to these treatments. This effect may be related to the transformation of melanoma cells into a mesenchymal-like state driven by *TCF4*, as well as the inhibition of melanogenesis and antigen presentation pathways, thereby promoting immune evasion. This suggests that intratumoral heterogeneity is a major contributor to resistance to immunotherapy, as it endows tumors with enhanced functionality and adaptability 12. However, our understanding of the origin, developmental trajectory, and current status of *TCF4*-positive melanoma cells and whether they possess specific pathways for immune-cell communication remains limited.

Given the exceptionally low response rate of PM to immunotherapy and its aggressive clinical behavior, it serves as an ideal model to study immunotherapy resistance, especially given its increasing incidence in recent years 13, 14. Our study identified a distinct evolutionary trajectory of *TCF4*-positive melanoma cells within a large sample of PM cells. Cell communication analysis revealed that melanoma subgroups at the terminal stage of this cellular fate exhibit the most extensive interactions among melanoma subpopulations and engage most frequently with immune cells. Furthermore, we determined their current state using tissue cell type scoring and traced their origin using pseudotime analysis. We found that these cells originate from a melanoma subgroup with the strongest stem cell capabilities, differentiating towards a neural direction through an intermediate state. Currently, these cells are in the process of differentiating towards Schwann cells, with observed decreases in gene expression related to pigment functions (*MLANA*, *MITF*, *TYR*, *PMEL*). In addition to increased expression of *TCF4*, we also found that this subgroup specifically expresses the genes *ECRG4* and *MPZ*, as well as increased levels of the transcription factors *HMGA2*. Based on these findings, we have named the distinct melanoma subgroup as Schwann-like melanoma cell subgroup and validated its spatial distribution through multiplex immunohistochemistry. Our results indicate that the proportion of Schwann-like melanoma cells increases with tumor progression and is associated with poorer prognosis. This explains why the PM has a lower immune response rate and a worse prognosis. Through this research, we hope to deepen the understanding of the development mechanism of melanoma and provide new perspectives and strategies for the clinical phenomenon of immunotherapy resistance.

## Methods

### Ethics approval

All clinical samples were collected with the explicit consent of the participants for research purposes. This study was conducted following the guidelines of the Declaration of Helsinki and was approved by our hospital's Ethics Committee (approval number 2022[304]). Informed consent was obtained from all participants to publish the relevant clinical data.

### Preparation of single-cell suspensions

We obtained 20 PM samples (experimental group) and seven samples of normal skin adjacent to benign plantar nevi (control group) from our hospital's Department of Dermatology. These samples were immediately placed on ice in MACS Tissue Storage Solution (catalog number: 130-100-008; Miltenyi Biotec, Bergisch Gladbach, Germany) as soon as they were procured from the operating room. The samples were then promptly transported to the laboratory within 30 min. Sections of the entire skin layer from the melanoma tissues, approximately 1.5×1.5 cm in size, were washed thrice with phosphate-buffered saline (PBS). The tissues were digested with 0.125 mg/mL trypsin solution at 37 °C for 15 min. The tissues were then rinsed with PBS containing 2% fetal bovine serum (FBS) and collected in a centrifuge tube, followed by centrifugation at 1000 rpm for 3 min. After discarding the supernatant, the cells were resuspended in PBS containing 2% FBS. The remaining undigested tissue pieces were finely minced and treated with a digestive mixture containing 2 mg/mL collagenase IV, 2.5 mM CaCl_2_, 0.1 mg/mL elastase, 2% FBS, and 10ug/mL DNA enzymes (catalog number: DN25-100MG, Sigma). The tissue mixture was shaken for 30 min to aid digestion.

Subsequently, the cells were centrifuged and resuspended in PBS containing 2% FBS. Finally, the suspension was sequentially strained through 100 µm and 40 µm cell filters, ensuring the complete collection of the sample volume. Cell quality was evaluated using the Countstar cell analysis system (Countstar Rigel S2; Alit Biotech, Shanghai, China) following cell centrifugation and resuspension. Cell viability was assessed in a single step using the trypan blue exclusion method facilitated by the Countstar device and its integrated intelligent image recognition technology. The samples were processed using the 10x Genomics protocol if cell viability exceeded 80%.

### Single-cell capture, library construction, and sequencing

We prepared the cell suspension (with a density of 600-800 viable cells per µL as determined by Countstar) for loading onto the Chromium Single Cell Controller (10x Genomics) using the Single-cell 3′ Library and Gel Bead Kit V3.1 (catalog number: 1000075, 10x Genomics, Pleasanton, CA, USA) along with the Chromium Single Cell B Chip Kit (catalog number: 1000074, 10x Genomics). This process followed the manufacturer's instructions and aimed at creating single-cell gel beads within an emulsion. Individual cells were incubated in PBS containing 0.04% bovine serum albumin. Approximately 10,000 cells were introduced into each channel, targeting a recovery of approximately 10,000 cells. The cells were lysed, and the released RNA was tagged during reverse transcription within each gel bead-in-emulsion. Reverse transcription was performed using the S1000TM Touch Thermal Cycler (Bio-Rad, Hercules, CA, USA), set at 53 °C for 45 min, then 85 °C for 5 mins, and finally maintained at 4 °C. Subsequently, the cDNA was synthesized and amplified, and its quality was evaluated using Agilent 4200 (performed by CapitalBio Technology, Beijing, China). The cDNA libraries were sequenced using the Illumina NovaSeq 6000 system, ensuring a minimum sequencing depth of 100,000 reads per cell. This process used a paired-end 150 base pairs (PE150) approach to read the sequences (performed by CapitalBio Technology).

### Data transformation using Cell Ranger

We employed the Cell Ranger software (version 7.0.1, 10x Genomics) to process data obtained from the chromium single-cell platform. Initially, the cellranger mkfastq command was used to demultiplex the raw base call files generated by Illumina sequencers and convert them into the FASTQ format. Subsequently, the cellranger count step took over processing FASTQ files generated by cellranger mkfastq, responsible for the alignment, filtering, and precise counting of barcodes and unique molecular identifiers. Sequence alignment was performed using the human reference genome GRCh38 (2020-A). Through these steps, the Cell Ranger software effectively generated feature barcode matrices using chromium cell barcode technology and provided foundational data for subsequent analyses in this study. The Cell Ranger software was directly obtained from the 10x Genomics website.

### Single-cell sequencing analysis

We integrated and analyzed single-cell transcriptomic data from patients with melanoma and healthy individuals. For instance, we used R 15 language (version 4.1.2) and the Seurat 16 software package (version 4.0.5) for comprehensive single-cell data integration to analyze 20 samples from patients with melanoma. Each sample was preprocessed using SoupX 17 (version 1.6.1) for background RNA correction to ensure the accuracy and reliability of the data. In addition, we used Scrublet 18 (version 0.2.3) to predict and eliminate doublets. Cells that Scrublet failed to predict accurately but clearly expressed multiple cell identity markers were manually removed. After reading the corrected single-cell data through Read10X and merging all samples into a single dataset, we excluded cells with an inadequate (≤400) or excessive (≥5000) number of genes and those with a high mitochondrial proportion (≥10%), thus ensuring the inclusion of only high-quality cells in the analysis. Ultimately, 154,940 cells and 33,510 genes were analyzed. Subsequently, we normalized each cell and calculated the cell cycle using the SCTransform transformation to regress the effects of mitochondrial content and cell cycle factors. During the feature-selection phase, we selected 3,000 highly variable genes using the SelectIntegrationFeatures function. Furthermore, we used the PrepSCT integration function to prepare a list of single-cell transcriptomic data objects standardized by SCTransform, laying an efficient and effective foundation for subsequent data integration. Each Seurat object underwent principal component analysis (PCA). We determined the integration anchors using the FindIntegrationAnchors function based on reciprocal PCA methods. Finally, we completed data integration using these anchors with the IntegrateData function. After integration, PCA was applied to the data to reveal and understand the main characteristics and trends. We then utilized the uniform manifold approximation and projection (UMAP) algorithm for further dimensionality reduction, clearly demonstrating cell relationships and distributions. Subsequently, we performed a clustering analysis on the cells by applying the FindNeighbors and FindClusters functions and setting the resolution parameter to 0.1, enabling us to identify and distinguish different cell populations in the data. To visually represent these findings, we generated UMAP scatter plots using the DimPlot function, which clearly displayed the clustering results and revealed the spatial distribution of cell subgroups. Furthermore, we used the FindAllMarkers function to identify differentially expressed genes (DEG) in each cell subgroup, thereby providing a deeper understanding of the biological characteristics of these subgroups. We precisely annotated each cell subgroup using molecular markers by combining previously collected cell type-specific genes. The cell identity-specific genes included: endothelial cells (*PECAM1*, *VWF*, *CDH5*, and *SELE*), melanocytes or melanomas (*MLANA*, *MITF*, *TYR*, and *PMEL*), T cells (*CD247*, *TRAC*, *TRBC2*, *CD4*, *CD8A*, and *CD8B*), pericytes (*ACTA2*, *TAGLN*, *RGS5*, and *NOTCH3*), keratinocytes (*IVL*, FLG, *KRT1*, *KRT10*, *KRT5*, and *KRT14*), fibroblasts (*FGF7*, *PDGFRA*, *PDGFRB*, *DCN*, and *LUM*), monocytes and macrophages (*CD14*, *S100A12*, *CLEC10A*, *CD68*, *CD80*, *CD86*, *CD163*, and *MRC1*), mast cells (*TPSB2*, *FCER1A*, *TPSAB1*, and *CMA1*), neutrophils (*ITGAM*, *FCGR3A*, *CD177*, and *CEACAM8*), skin appendage keratinocytes (*EPCAM*, *KRT18*, *KRT7*, and *KRT19*), lymphatic endothelial cells (*PROX1*, *LYVE1*, and *FLT4*, *PDPN*), B cells (*CD19*, *MS4A1*, *CD79A*, and *CD79B*), and Schwann cells (*NGFR*, *MPZ*, *MAL*, and *PMP22*).

### Cell communication analysis

In this study, we focused on an in-depth analysis of cell-cell communication in PM tissues. Using the CellChat 19 (version 1.1.3) tool, we explored the interactions between various cell types within the tissue by identifying significant ligand-receptor pairs. This process included analyzing the expression of ligand genes and the corresponding receptor genes in specific cell groups, thereby revealing the cell communication network in PM. This approach enabled us to quantitatively assess and understand the cell-cell communication characteristics of this specific melanoma type.

### CNV analysis

We employed the CopyKAT 20 (Copy Number Analysis of Tumors, version 1.0.8) technique, an advanced method integrating Bayesian analysis aimed at whole-genome aneuploidy analysis on a single-cell level across various samples. This method has a resolution of approximately 5 MB and can distinguish melanoma cells from normal melanocytes and identify tumor subclonal structures. For accurate DNA copy number estimation, we stipulated that each chromosome must contain at least five genes, using the gene symbol (“S”) to identify gene IDs. Furthermore, to ensure the precision and reliability of the analysis, each copy number analysis segment was required to include at least 25 genes. In addition, we set the segmentation parameter KS.cut to 0.1 to enhance the sensitivity of the analysis. Notably, increasing the KS.cut value decreases the sensitivity of the analysis.

### Melanoma subgroup analysis

During the in-depth analysis of melanoma subgroups, we used predictive data from CopyKAT to select aneuploid melanoma subgroups. A more refined dimensional reduction and clustering analysis was performed on the selected subgroups within the integrated dataset. By comprehensively considering aspects such as gene expression characteristics, functional enrichment analysis results, the cell's differentiation potential, and characteristics at various stages from initiation through transition to the terminal state, we could accurately identify five distinct melanoma cell subgroups, each characterized by unique functional properties.

### Mapping cell subpopulations

Using the MapQuery function in the Seurat package, a target query dataset was mapped to a reference dataset. This method has three core functions, TransferData, IntegrateEmbeddings, and ProjectUMAP, which are typically used for mapping query data to reference data. TransferData was used to transfer cell-type labels and estimate antigen detection technology values, IntegrateEmbeddings was used to integrate reference data by correcting the query's projected low-dimensional embeddings, and ProjectUMAP was used to project the query data onto the UMAP structure of the reference data. The MapQuery function enabled us to map normal cell types to tumor tissue cell types, thereby exploring the differences and similarities between the tumor and normal tissues.

### GSEA-GOBP analysis

The present study used the GSEA-GOBP enrichment analysis method in ClusterProfiler 21 (version 4.2.0) to investigate the biological characteristics of melanoma cell subgroups at the single-cell level. Using the FindMarkers function in the Seurat toolkit, we successfully identified DEG between various melanoma cell subtypes and other subgroups. During this process, we set the parameter logfc.threshold to 0 and constructed a gene list containing all DEG arranged in descending order of their average log2 fold change (avg_log2FC). Subsequently, this gene list was analyzed using the gseGO function in ClusterProfiler. Our analysis focused on the Biological Process category using the human gene database (org.Hs.eg.db) and Entrez Gene ID as the unique identifier for genes (keyType = "ENTREZID"). We set nPermSimple to 10000 and adjusted minGSSize to 10 and maxGSSize to 500, based on the actual size of the gene sets, to ensure the accuracy and robustness of our analysis. Statistical significance was set at a p-value of 0.05, and the Benjamini-Hochberg method was used to correct p-values in multiple tests. Finally, we selected gene pathways with p.adjust <0.05 and normalized enrichment score values ranked in the top 10 for each subgroup for the presentation of our results.

### Gene set variation analysis

We used the AverageExpression function of the Seurat software package to calculate the average gene expression matrices for Schwann cells, melanocytes, diploid melanoma cells, and aneuploid melanoma cells. Subsequently, gene sets associated with human biological processes were selected from the Molecular Signatures Database (MSigDB) 22. We meticulously scored these gene sets using the GSVA 23 method (version 1.42.0) to assess the pathway activities in different cell subgroups. Specifically, we conducted a detailed analysis of gene sets associated with pigment synthesis and neural development using MSigDB. We performed an in-depth quantitative assessment of the molecular characteristics and functional states of these cell subgroups.

### Cell subgroup development potential and differentiation state assessment

CytoTRACE 24 has been extensively used to predict the differentiation state of cells in the single-cell transcriptomes of various cell types, tissues, lineages, and species. It is based on the simple yet powerful principle of determining the number of detectably expressed genes per cell (gene counts). By analyzing gene counts, CytoTRACE can assess the developmental potential and degree of cell differentiation, revealing their states and trajectories during development. This study used CytoTRACE (version 0.3.3) to predict the differentiation potential and degree of the PM cell subgroups. We mapped the CytoTRACE scoring results onto UMAP and used boxplots to summarize the median and distribution of the CytoTRACE values across different phenotypes.

### Cellular RNA velocity analysis using scVelo

scVelo 25 (version 0.2.4), an advanced analytical tool for studying cell differentiation, development, and other dynamic states at the single-cell level, possesses sophisticated dynamic modeling and analytical capabilities that can reveal subtle changes in intracellular RNA velocity. scVelo can estimate RNA velocity and identify key driver genes, infer potential timelines for the sequence of transcriptomic events, accurately calculate the rates of transcription, splicing, and degradation, and apply statistical tests to distinguish between different kinetic regimes. We used the scVelo technology to explore variations in RNA velocity among different melanoma subtypes.

### Calculating transition state scores

Capybara 26 (version 0.0.0.9) is designed to precisely measure and distinguish cell identity and transformation processes. It specializes in identifying and categorizing the discrete states of cells and their intermediate “hybrid” states and offers an effective method for quantifying the dynamics of cell fate transitions. The uniqueness of this tool lies in its ability to continuously track changes in cell identity at the single-cell level. Capybara can recognize various identities and compute a metric called the “transition state score” for cells exhibiting multiple identity characteristics. We used Capybara to analyze the transition state scores of five functionally diverse melanoma subtypes to identify the intermediary transition states of these tumor subgroups.

### CellRank analysis

CellRank 27 (version 1.5.1) is designed to study dynamic biological processes such as development, regeneration, cancer, or cellular reprogramming. It analyzes multidimensional single-cell genomic data, including RNA velocity and developmental potential, to accurately infer cellular developmental trajectories and fate probabilities and identify key genes driving these processes. CellRank precisely identifies the initial and terminal states of cellular development using Markov chains to describe random transitions between cellular states and infers intermediate transitional states. This study used CellRank to deduce the developmental trajectories of the melanoma subgroups.

### Transcription factor regulatory network analysis

pySCENIC 28 (version 0.12.1) is an efficient computational tool based on the Python (version 3.7.12) platform designed to infer gene regulatory networks from scRNA-seq data. Our study used pySCENIC to investigate transcription factor regulatory networks in specific cell subgroups, thereby gaining a deeper understanding of gene regulation patterns within these cell subgroups.

### Tissue-cell type inference

WebCSEA 29 provides an efficient interface for querying gene sets within a comprehensively collected tissue-cell type expression characteristic database. This tool enables cell-type-specific analysis and summarizes data based on the human organ system, identifying the top-ranked tissues and common cell types. This study used the Findallmakers function to analyze the differential genes between the five melanoma subgroups, with parameters set to min.pct = 0.25 and logfc.threshold = 0.25. After filtering with p_val_adj <0.05, the top 50 differential genes ranked by avg_log2FC were analyzed using WebCSEA to determine the tissue cell types associated with the melanoma subgroups.

### Spatial transcriptomics sequencing and analysis

Tumor tissue samples from PM patients were first washed with 1× PBS buffer and then rapidly frozen in isopentane cooled with liquid nitrogen. Tissue samples were then embedded in OCT compound, placed on dry ice for preservation and finally stored at -80°C. Tissue sections of 10 μm thickness were placed on pre-cooled tissue optimisation and gene expression slides (BMKMANU S1000). Tissue samples underwent fixation, nuclear staining, H&E staining and imaging according to the manufacturer's standard protocol. Upon completion of the reverse transcription reaction, the cDNA library constructed with spatial information underwent quality control and was sent to the Illumina NovaSeq 6000 platform for 150 bp paired-end sequencing. The sequencing data were then used to analyse the spatial distribution characteristics of the melanoma subpopulations. Using Seurat's anchor-based integration workflow, we further scored the transcriptomic features of melanoma subpopulations and exhausted T cells on the spatial transcriptome, showing their spatial distribution patterns.

### Multiplex immunofluorescent staining

We performed multiplex immunohistochemical staining with MLANA (cat 60348-1-Ig, 1:200), MITF (cat ab303530, 1:5000), CXCL1 (cat GTX03810, 1:200), ECRG4 (cat GTX87799, 1:200), NGFR (cat ab52987, 1:200), MPZ (cat ab183868, 1:500), TCF4 (cat ab223073, 1:200) and HMGA2 (cat ab97276, 1:200) on 4-µm formalin-fixed, paraffin-embedded tumor sections. The PANO 7-plex immunohistochemistry (IHC) kit (product number 0004100100; Panovue, Beijing, China) was used for multiplex IHC staining, using antibody combinations specific to Figures 4 and 8. After incubation with each primary antibody, horseradish peroxidase-conjugated secondary antibody was added, and tyramide signal amplification (TSA) was performed. After each TSA treatment, the slides were subjected to microwave processing. Once all human antigens were labeled, the nuclei were stained with 4′-6′-diamidino-2-phenylindole (DAPI, SIGMA-ALDRICH). Automated staining of 30 different malignant melanoma tissues was performed using Leica Bond RX with staining antibodies against MITF (cat ab303530), CXCL1 (cat GTX03810), NGFR (cat ab52987), and ECRG4 (cat GTX87799).

### Survival analysis

We performed survival analyses in 30 patients with melanoma with varying degrees of malignancy. We used the survival (version 3.2) and survminer (version 0.4.9) tools from the R package to accurately determine the survival times and statuses of the patients. Accordingly, we constructed detailed survival curves.

### Chromium single cell multiome ATAC + gene expression sequencing and analysis

Raw sequencing data were converted to fastq format using cellranger-arc mkfastq (10x Genomics, version 2.0.1). scATAC-seq reads were aligned to the GRCh38 (hg38) reference genome and quantified using cellranger-arc count (10x Genomics, version 2.0.1). The feature barcode matrices for both RNA-seq and ATAC-seq data were integrated using the Seurat (version 5.1.0) and Signac 30 (version 2.4.0) packages, following the standard protocol described in the manufacturer's guidelines. Per-cell quality control metrics were calculated using both snRNA-seq and scATAC-seq data, and cells identified as outliers based on these metrics were excluded. To ensure data integrity, RNA contamination in snRNA-seq data was corrected using SoupX (version 1.6.1) and potential doublets were removed using Scrublet (version 0.2.3). In addition, we manually excluded doublets that were not detected by Scrublet but had overlapping expression of cell identity markers. High quality cells were retained by applying stringent quality control filters, including ATAC fragment counts below 40,000 and above 1,000, RNA UMI counts below 30,000 and above 500, fewer than 6,000 detected genes, nucleosome signal below 1, TSS enrichment score above 1, and mitochondrial gene percentage below 15%. Melanoma subgroups were identified based on the expression of established markers, including *MLANA*, *MITF*, *TYR* and *PMEL*. After applying combined ATAC and RNA quality filters, a total of 9,007 cells with high quality measurements in both modalities were retained for downstream analysis. We constructed a weighted nearest neighbour (WNN) graph that integrates information from both RNA-seq and ATAC-seq modalities. This graph was used for UMAP visualisation and clustering analysis. Cell clusters were annotated based on the expression of key marker genes, including *MLANA*, *MITF*, *CXCL1*, *EREG4*, *NGFR*, *MPZ*, *HMGA2*, and *TCF4*. Using Signac, we visualised the chromatin accessibility traces for *HMGA2* and *TCF4*, together with gene annotations, peak coordinates, genomic links and fragment positions.

### QuSAGE

To investigate the signalling characteristics and molecular heterogeneity of melanoma subgroups in relation to the mesenchymal-like melanoma described by the Pozniak *et al.*, we performed Quantitative Set Analysis for Gene Expression 31 (QuSAGE, version 2.28.0). Gene sets were derived using the FindAllMarkers function to identify differentially expressed genes (DEGs) within each cell subgroup with an adjusted p-value (p_val_adj) < 0.05, selecting the top 10 genes ranked by average log2 fold change (avg_log2FC).

### Cell line and culture conditions

The human plantar melanoma cell line LM-MEL-45 was generously provided by Dr Jun Guo (Peking University Cancer Hospital). Cells were maintained in Dulbecco's modified Eagle's medium (DMEM) supplemented with 10% fetal bovine serum (FBS) and 1% penicillin-streptomycin and cultured at 37°C in a humidified incubator with 5% CO2.

### siRNA transfection

Cells were divided into three groups: (1) siHMGA2-transfected cells, (2) siTCF4-transfected cells and (3) a negative control group transfected with non-targeting siRNA. Transfection was performed using Lipofectamine 3000 (Invitrogen, USA) according to the manufacturer's instructions. Briefly, cells were seeded in 6-well plates and transfected at 60-70% confluence. After 48 hours of transfection, cells were harvested for downstream functional assays. Cells were transfected with small interfering RNA (siRNA) targeting the HMGA2 gene (sense: GGAUGAUGCUAUUCAUGUUTT; antisense: AACAUGAAUAGCAUCAUCCTT), the TCF4 gene (sense: AGUCCCUCUAAAGCAGCUCAATT; antisense: UUGAGCUGCUUUAGAGGGACUTT) or negative control RNAi, all purchased from KeyGen BioTECH (Beijing, China). Transfections were performed using Lipofectamine 3000 transfection reagent (L3000015, Invitrogen) according to the manufacturer's protocol.

### Cell proliferation assay

Cell proliferation was assessed using the Cell Counting Kit-8 (CCK-8, CK04-500, Dojindo, Japan) according to the manufacturer's protocol. Briefly, cells from each group (siHMGA2, siTCF4 and control) were seeded in 96-well plates at a density of 2 × 10³ cells per well and incubated for 0, 24, 48, 72, 96 and 120 hours. At each time point, 10 µL of CCK-8 reagent was added to each well and the plates were incubated for a further 2 hours at 37°C. Absorbance was measured at 450 nm using a microplate reader (BioTek, USA).

### Scratch assay for cell migration

Cell migration ability was assessed using a scratch assay. Cells from each group (siHMGA2, siTCF4 and control) were seeded in 6-well plates and grown to 90% confluence. A uniform scratch was made in the cell monolayer using a sterile 200 µL pipette tip. After gentle washing with phosphate-buffered saline (PBS) to remove detached cells, fresh medium was added. Images of the scratch were taken at 0, 24 and 48 hours using an inverted microscope (Nikon, Japan). Migration distance was quantified using ImageJ software (NIH, USA).

### RT-qPCR

Total RNA was isolated from cultured cells using TRIzol (15596026CN, Invitrogen), followed by reverse transcription into single-stranded cDNA using a first-strand cDNA synthesis kit (F0202-100T, LABLEAD) according to the manufacturer's instructions. RT-PCR was performed using PowerUp™ SYBR™ Green Master Mix (A25742, Thermo Fisher) on a PCR detection system (Step ONE, Applied Biosystems). The relative mRNA expression levels of the target genes were quantified using the 100x2^-ΔΔCT method. Sequences of RT-qPCR primers are given in Table S4.

### Bulk RNA sequencing

Total RNA was extracted from LM-MEL-45 cells using TRIzol (15596026CN, Invitrogen). Strand-specific mRNA-seq libraries were constructed and sequenced on the Illumina NovaSeq 6000 platform, generating 150 bp paired-end reads. Principal component analysis (PCA) was performed using the DESeq2 32 (version 0.2.3) R package. Differential expression analysis was performed using DESeq2 after normalisation of read counts. DEGs were identified as genes with |log2(fold change)| > 1 and a Benjamini-Hochberg adjusted p-value ≤ 0.05. The identified DEGs were then submitted to the Gene Ontology Biological Process (GOBP) database for enrichment analysis. In addition, the clinical cohort included 68 plantar melanoma patient samples, which were primarily used to analyse the relationship between *HMGA2* expression levels and patient prognosis.

### Statistics analysis

T-tests were used for pairwise comparisons of cell distributions among *in situ*, invasive, and normal melanoma samples. Similarly, t-tests were used for pairwise comparisons of melanoma subgroup distributions among normal, *in situ*, invasive, and metastatic samples in mIHC. All statistical analyses and visual displays were performed using R software. The statistical significance level was set at P < 0.05. Unless otherwise noted, two-tailed tests were used. P values > 0.05 = ns, P values ≤ 0.05 = *, P values ≤ 0.01 = **, P values ≤ 0.001 = ***.

## Results

### The key role of the MM4 subgroup in tumor-immune cell communication

**Figure 1:**
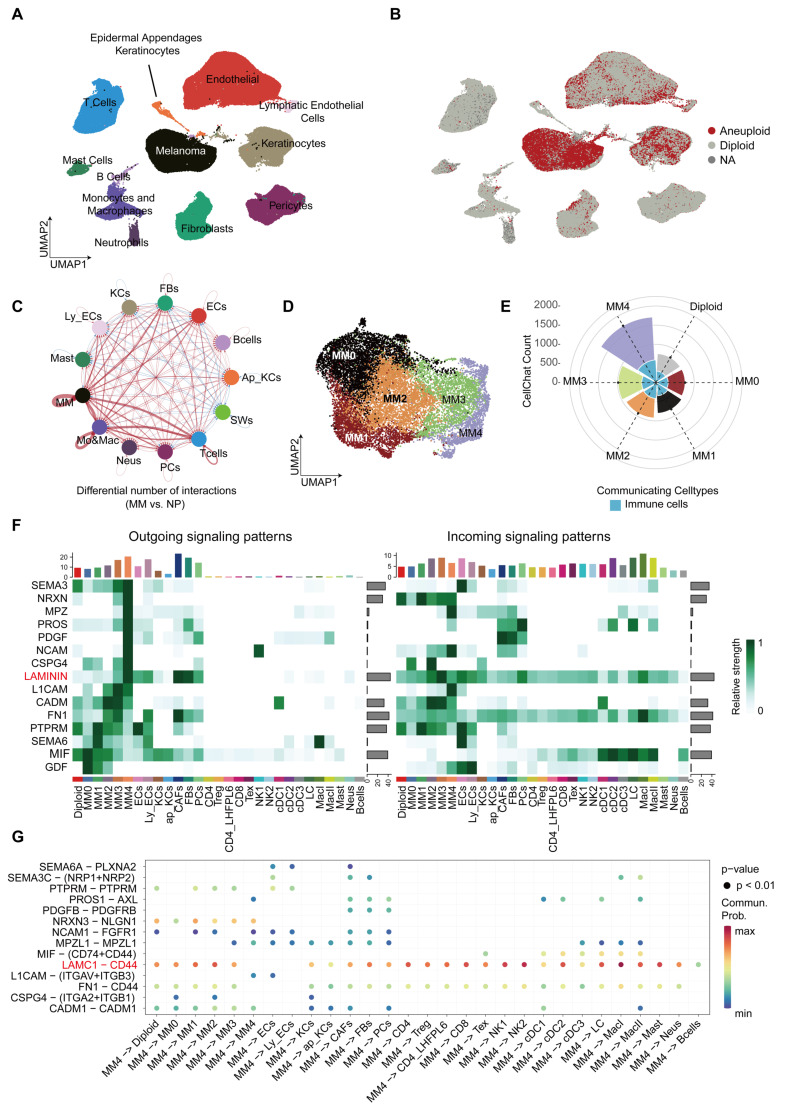
** Discovery of the MM4 melanoma subgroup and its intercellular communication properties.** A. UMAP displays the main cell types in the PM tumor microenvironment. B. Copykat analysis of melanoma samples for copy number variations. Red represents aneuploidy, gray represents diploidy, and NA represents unpredictable cells. C. Circle plot showing the differential number or strength of interactions between different cell populations in PM and normal plantar skin. Red (or blue) edges represent increased (or decreased) signaling in melanoma compared to normal tissue. Melanoma Cells (MM), Keratinocytes (KCs), Fibroblasts (Fbs), Endothelial Cells (ECs), B cells (Bcells), Epidermal Appendages Keratinocytes (Ap_KCs), Schwann Cells (SWs), Lymphatic Endothelial Cells (Ly_ECs), Mast Cells (Mast), Monocytes & Macrophages (Mo&Mac), Neurons (Neus), Pericytes (PCs), T cells (Tcells). D. UMAP shows melanoma cell subgroups. E. The circular fan chart shows the total cell communication counts for each PM subtype as calculated using CellChat. The MM0 subgroup is shown in red, MM1 in black, MM2 in orange, MM3 in green, MM4 in purple, and diploid melanoma in gray. The area of light blue fan-shaped sections in the inner circle represents the total number of communications between each melanoma subtype and immune cells, including T cells, B cells, dendritic cells, monocytes, neutrophils, and mast cells. F. The heatmap illustrates the significant impact of the most critical signals on outgoing signaling within PM melanoma subgroups and shows the cell types receiving these signals along with the signal strength. We have selected ligand-receptor pathways with a signal strength greater than 0.8 for display. The color scale clearly shows the relative signal strength of pathways in different cell groups. The colored bar graph at the top aggregates and displays the total signal strength for each cell type, while the gray bar graph on the right aggregates and displays the total signal strength for each pathway. CD4: CD4+ T cells; Treg: Regulatory T cells; CD4_LHFPL6: CD4+ LHFPL6+ T cells; CD8: CD8+ T cells; Tex: Exhausted T cells; NK1, NK2: Natural killer cells subsets; cDC1, cDC2, cDC3: Dendritic cells subsets; LC: Langerhans cells; MacI, MacII: Macrophage subsets; Mast: Mast cells; Neus: Neutrophils. G. We filtered the most potent ligand-receptor pairs within the key signaling pathways in panel f to illustrate the communication between the MM4 subgroup and other cell types through these ligand-receptor interactions.

We performed single-cell RNA sequencing (scRNA-seq) analysis on samples from 20 patients with PM, obtaining a total of 154,940 cells. Through annotation, we identified 12 major cell types, including tumor cells, immune cells, and stromal cells (Figure 1A). In addition, we obtained a total of 56,918 cells from six normal samples, with specific annotation details shown in Figure S1A (available online). Detailed clinical and pathological data are presented in Table S1. Copy Number Karyotyping of Aneuploid Tumors 20 (CopyKAT) was used for individual patient analysis to differentiate between normal and malignant cells in the tumor microenvironment (Figure 1B). We identified 15,274 aneuploid tumor cells. Their copy number variation (CNV) heatmap is presented in Figure S1B (available online). The aneuploid cells showed higher malignancy and deeper tumor invasion (Figure S1C).

Through CellChat 19 analysis, we found that melanoma cells within the tumor microenvironment have a significantly enhanced ability to communicate with each other compared to melanocytes in normal tissue (Figure 1C). Further analysis of aneuploid tumor cells led to the identification of five distinct melanoma cell subgroups based on transcriptomic expression patterns (Figure 1D). We discovered that the MM4 subgroup had the highest communication frequency with other cell types among all tumor cell subgroups, particularly showing frequent interactions with immune cells (Figure 1E and Figure S1D-I). Importantly, the MM4 subgroup primarily signals through *SEMA3*, *NRXN*, *MPZ*, *PROS*, *PDGFR*, *NCAM*, *CSPG4*, and *LAMININ* (Figure 1F). Moreover, the LAMC1-CD44 axis serves as the major ligand-receptor pathway for communication between MM4 and immune cells (Figure 1G). In addition, MM4 broadly affects stromal cell types through the MPZL1-MPZL1 interaction (Figure 1G).

### MM4 subgroup evolution and status

**Figure 2:**
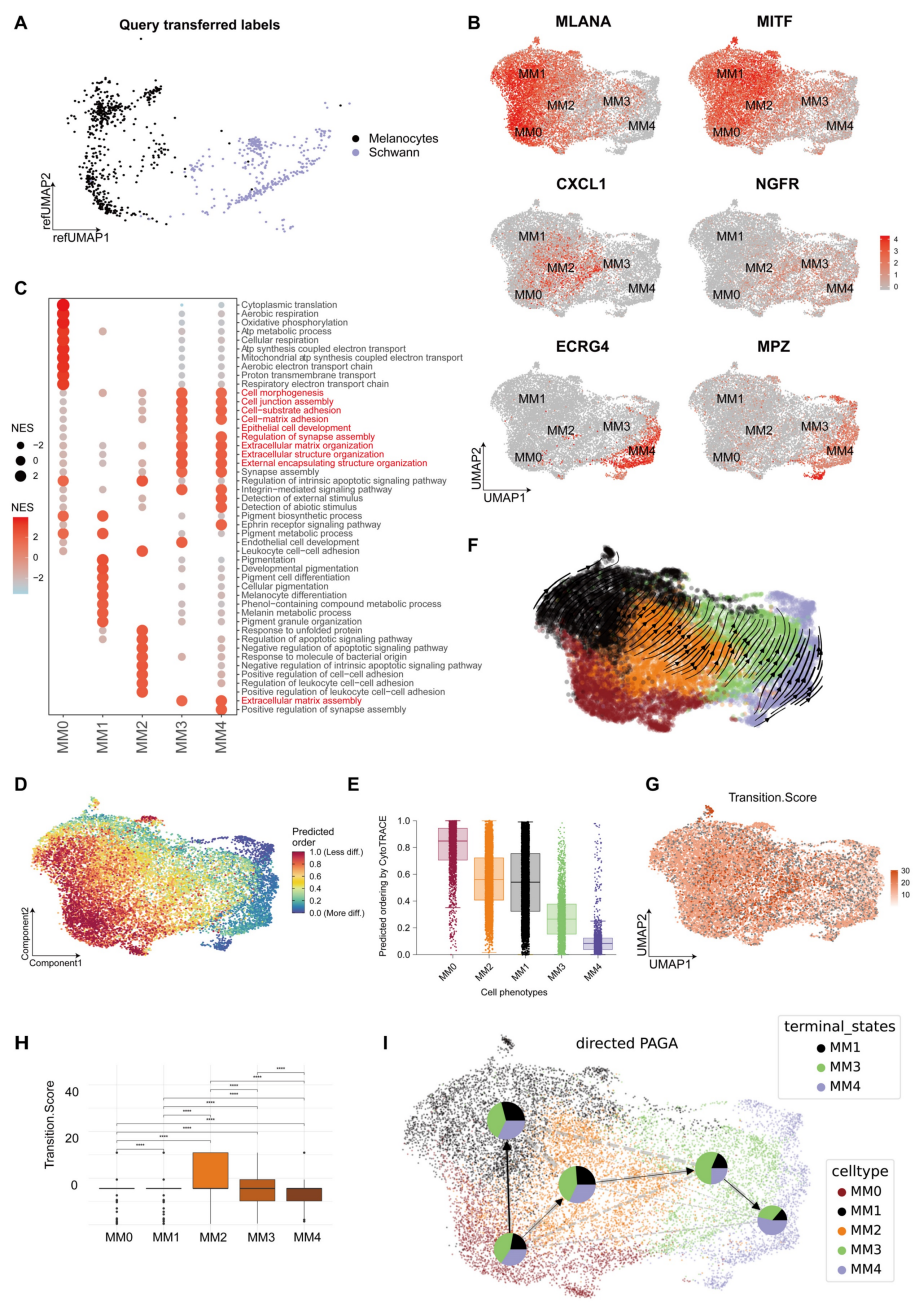
** Melanoma subgroup analysis.** A. UMAP shows the mapping of normal melanocytes and Schwann cells to the melanoma subgroups. B. The UMAP plot illustrates the distribution of markers associated with melanoma subgroups, including *MLANA*, *MITF*, *CXCL2*, *NGFR*, *ECRG4*, and *MPZ*. C. In the melanoma subgroups, Gene Set Enrichment Analysis-Gene Ontology Biological Process (GSEA-GOBP) analysis selected gene pathways with p.adjust values <0.05 and ranked them in descending order of normalized enrichment score (NES) values, displaying the top 10 enriched gene pathways. The sizes and colors of the circles represent the magnitudes of the NES values. D. Cellular Trajectory Reconstruction Analysis using Gene Counts and Expression (CytoTRACE) displays the differentiation potential of the PM subgroups, with red indicating a strong differentiation potential and blue indicating a weak potential. E. Boxplots summarizing the median and distribution of CytoTRACE values for each phenotype. F. scVelo was used to analyze RNA velocity in the plantar melanoma (PM) subgroups, with arrows indicating the direction of RNA velocity. G. Capybara was used to assess the transition state scores of melanoma subgroups. H. Boxplots were used to compare the differences in transition state scores between melanoma subgroups (t-test). I. CellRank was used to analyze the pseudo-temporal developmental trajectory of melanoma subgroups, with pie charts showing the probability of each subgroup transitioning to a terminal state and arrows indicating the predicted developmental fate trajectories.

PM shows a mixed state of pigment and neural function. The Seurat mapping analysis revealed an alignment of normal melanocytes with the terminal stages of the MM0 and MM1 subgroups. However, Schwann cells aligned with the later stages of the MM3 and MM4 subgroups (Figure 2A). Furthermore, melanoma cells simultaneously expressed characteristic markers associated with melanocytes and neurons (Figure 2B and S2).

We annotated these subgroups using Gene Set Enrichment Analysis-Gene Ontology Biological Process (GSEA-GOBP) 23, highlighting their unique transcriptomic profiles (Figure 2C). MM0 was characterized by energy metabolism processes, including cytoplasmic translation and oxidative phosphorylation; MM1 was closely associated with pigment production processes; MM2 was primarily associated with immune and anti-apoptotic regulatory pathways; MM3 exhibited functions associated with cell adhesion and extracellular matrix, suggesting a potential role in tumor dissemination; and MM4 shared functional characteristics with MM3 but uniquely showed sensitivity to external and abiotic stimuli, reflecting an adaptation to environmental pressures (Figure 2C).

We used various bioinformatics analysis software to predict the initial, intermediate, and terminal states of melanoma and investigate the origins and evolutionary paths of MM4. Initially, we quantified gene expression in scRNA-seq data using Cellular Trajectory Reconstruction Analysis using Gene Counts and Expression (CytoTRACE) 24 technology to assess cell stemness and differentiation potential (Figure 2D and 2E). The analysis indicated that cells in the MM0 subgroup exhibited the highest stemness, potentially representing the initial state of melanoma subgroups. However, a gradual decline in stemness and a corresponding decrease in differentiation potential were observed from the MM2 to MM4 subgroups. Furthermore, we applied dynamic modeling with scVelo 25 to track the cell state evolution. The cell velocity curves showed that the cells originated from the MM0 subgroup and gradually differentiated into several melanoma subgroups (Figure 2F). In addition, we used the Capybara 26 computational platform for a quantitative assessment of the transitional states of cell populations, where the MM2 subgroup was outstanding in transitional state scoring, suggesting it as a key intermediate state in cellular fate transitions (Figure 2G and 2H). Finally, we conducted a pseudo-temporal analysis using Cellrank 27 to clarify the relationships among different melanoma subgroups 27, integrating gene expression and cell velocity data. We deduced that the melanoma cell subgroups originated from MM0 and diverged into two distinct pathways: one leading to the pigmentary MM1 subgroup and the other progressing through MM2 as a crucial intermediate state, eventually evolving into the MM3 and MM4 subgroups as terminal states (Figure 2I).

### MM4 melanoma subgroup show Schwann cell-like characteristics

**Figure 3:**
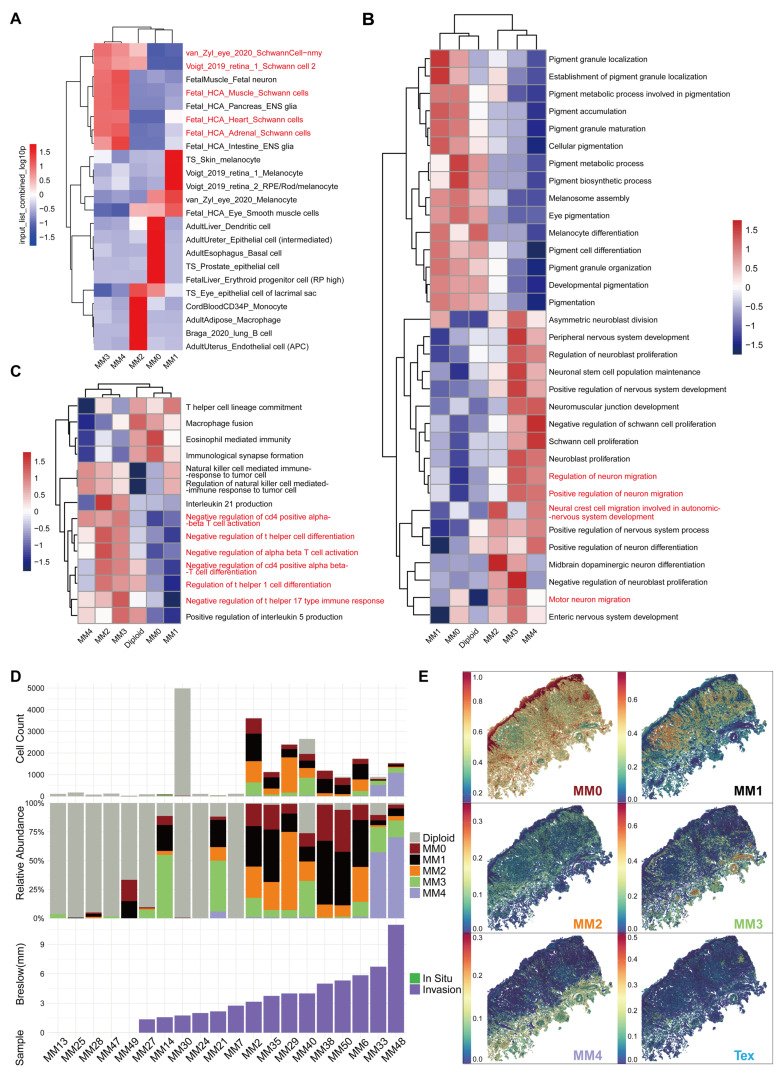
** Tissue cell identity prediction and characteristics of melanoma cell subgroups.** A. Web-based cell-type-specific enrichment analysis (WebCSEA) analysis was performed to predict the corresponding tissue cell types of various melanoma cell subgroups. The heatmap displays the top five primary tissue cell types corresponding to each melanoma cell subgroup, with different colors representing the input_list_combined_log10p values from the WebCSEA analysis. B. The GSVA analysis quantified the differences in the activity of the pigment synthesis and neural-related pathways between the melanoma subgroups, where red represents increased pathway activity and blue represents decreased pathway activity. C. The GSVA analysis quantified the differences in immune-related pathway activity between melanoma subgroups, with red indicating increased pathway activity and blue indicating decreased pathway activity. D. Bar charts show the number of melanoma cells in each sample (top), with gray, red, black, yellow, green, and purple representing diploidy, melanoma-initiating cell (MM0), pigmented melanoma (MM1), intermediate transitional state melanoma (MM2), Schwann-like melanoma precursor (MM3), and Schwann cell-like melanoma (MM4) subgroups, respectively. Stacked proportion charts display the proportion of each melanoma cell subgroup in samples (middle). Bar charts show the Breslow invasion depth of tumors in each sample (bottom), with green representing *in situ* melanoma and purple representing invasive melanoma. E. The SpatialFeaturePlot shows the prediction scores for each spot for each melanoma subgroup and exhausted T cells (Tex) in spatial transcriptomics sequence data.

We used the Web-based cell-type-specific enrichment analysis (WebCSEA) 29 tool to predict the corresponding tissue cell types in various melanoma cell subgroups (Figure 3A). The results revealed that the MM0 subgroup was phenotypically similar to erythroid progenitor cells, suggesting that it may be at an earlier stage of development. The MM1 subgroup showed melanocyte characteristics, whereas the MM2 subgroup resembled immune cells, such as monocytes and macrophages, with a significant upregulation in the major histocompatibility complex (MHC)-II gene expression (Figure S3). Furthermore, the MM3 and MM4 subgroups, especially the MM4 subgroup, were more closely associated with the Schwann cell phenotype, which demonstrated the specific expression of *MPZ*, further emphasizing its similarity to mature myelin Schwann cells (Figure 2B). Given that the pseudotime trajectory of the MM3 subgroup is more consistent with an intermediate state of Schwann-like differentiation rather than mature neuronal characteristics, we propose to name MM3 the "Schwann-like melanoma precursor subgroup" to highlight its critical transitional role in the process of Schwann-like differentiation (Figure 2B, 2I and 3A).

Through Gene Set Variation Analysis-Gene Ontology Biological Process (GSVA-GOBP), we observed that during the transition of melanoma to a Schwann-like fate, pigment function gradually decreased, neuronal-related functions and migratory capacity significantly increased, and a negative regulatory state of immune function was evident (Figure 3B and 3C). Furthermore, the analysis of clinical information indicated an increasing trend in cell group diversity with greater invasion depth of malignant melanoma (Figure 3D). This was particularly evident in two patients with a Breslow thickness ≥ 6 mm, where the MM4 subgroup was the most predominant. Mapping the melanoma subgroups to the spatial transcriptomic data of a melanoma sample revealed that the MM4 subgroup showed the greatest depth of invasion and was in the closest spatial proximity to exhausted T cells (Figure 3E). We further analyzed single-cell sequencing data from 20 cases to investigate the correlation between the proportion of melanoma subgroups and immune cell subgroups. The results showed that variations in MM4 abundance across different patients were more significantly correlated with the proportion of exhausted T cells (Figure S4).

Based on these findings, the five melanoma subgroups are defined as follows: melanoma-initiating cell (MM0), pigmented melanoma (MM1), intermediate transitional state melanoma (MM2), Schwann-like melanoma precursor (MM3), and Schwann cell-like melanoma (MM4).

### Spatial distribution characteristics of Schwann-like melanoma subgroups

**Figure 4:**
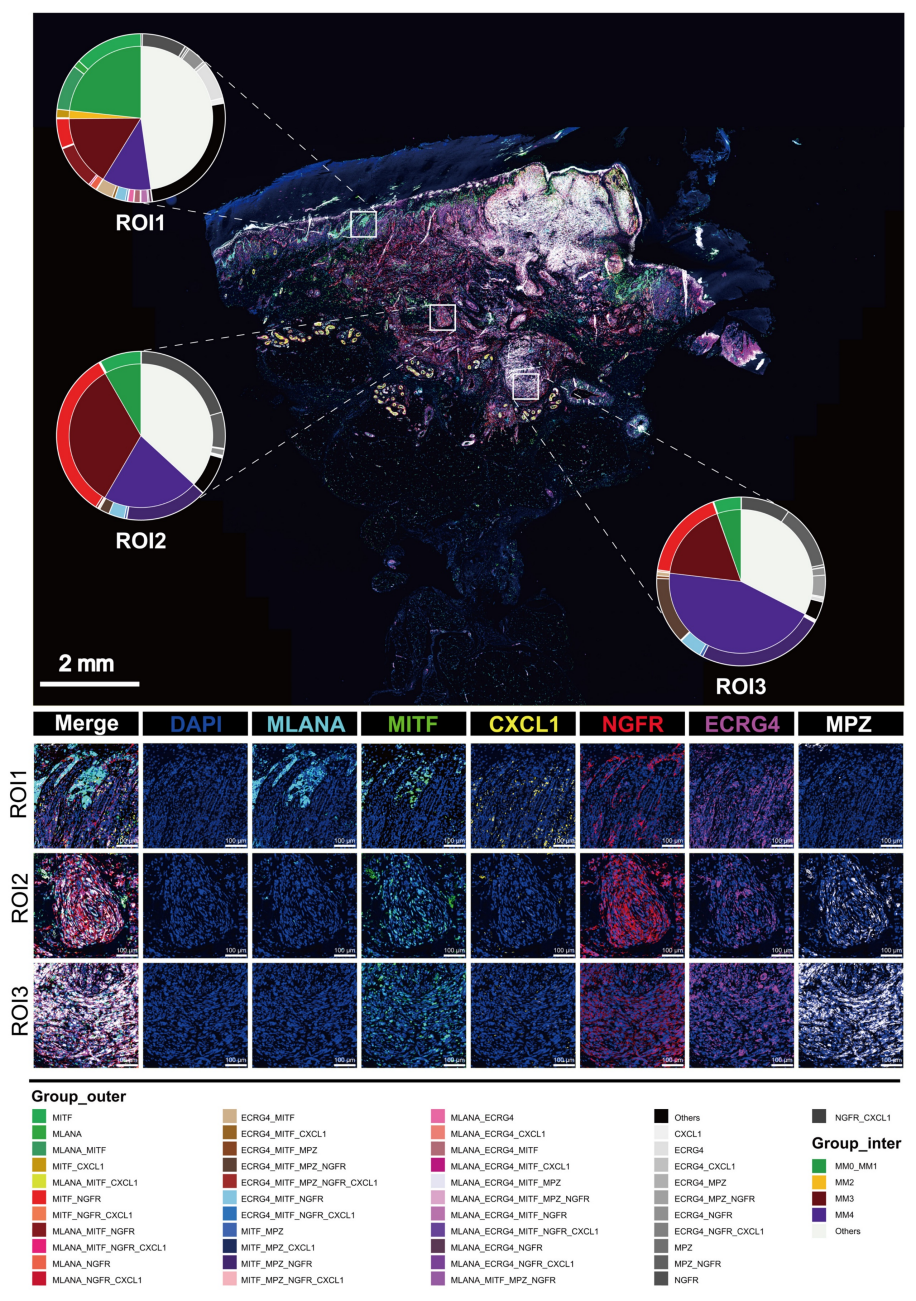
** Multiple immunohistochemistry spatial distribution of melanoma subgroups in invasive melanoma tissue.** We selected a case of invasive melanoma with a Breslow thickness of 6.73 mm. Three different areas from the epidermis to the dermis were selected to demonstrate the distribution of MLANA (cyan), MITF (green), CXCL1 (yellow), NGFR (red), ECRG (purple) and MPZ (White) antibodies using mIHC. 4′-6′-diamidino-2-phenylindole (DAPI; blue) indicates the cell nuclei. A double pie chart displays the staining results in different areas; the inner pie chart shows the melanoma-initiating cell (MM0), pigmented melanoma (MM1), intermediate transitional state melanoma (MM2), Schwann-like melanoma precursor (MM3), and Schwann cell-like melanoma (MM4) subgroups, whereas the outer ring corresponds to different antibody combinations. Other antibody combinations were categorized as “other” types.

To precisely map the spatial distribution of melanoma subgroups within the tissue, we performed mIHC analysis on an invasive melanoma specimen with a Breslow thickness of 6.73 mm (Figure 4), using identity markers identified by quantitative differential gene expression analysis (Figure 2B). Through mIHC staining of consecutive regions from the superficial to the deeper layers of the tumor, spanning three different fields of view, the spatial distribution patterns of melanoma cell subgroups within the tumor microenvironment were observed. The specific details of the mIHC antibody grouping are provided in the legend of Figure 4. In region 1, closest to the basal layer, the MM0 and MM1 subgroup had the highest proportion among the three views. In region 2, NGFR expression was enhanced, spreading throughout the tumor nest and signaling an increasingly evident trend toward a MM3 phenotype transformation. Delving deeper into regions 3, as the invasion of the melanoma nests intensified, the transformation to the MM4 subgroup was fully realized, making it the predominant cell type within the tumor (Figure 4 and S5). At this stage, the expression of NGFR, ECRG4 and MPZ was markedly enhanced (Figure 4).

### The Schwann-like melanoma subgroup status indicates a poorer patient prognosis

**Figure 5:**
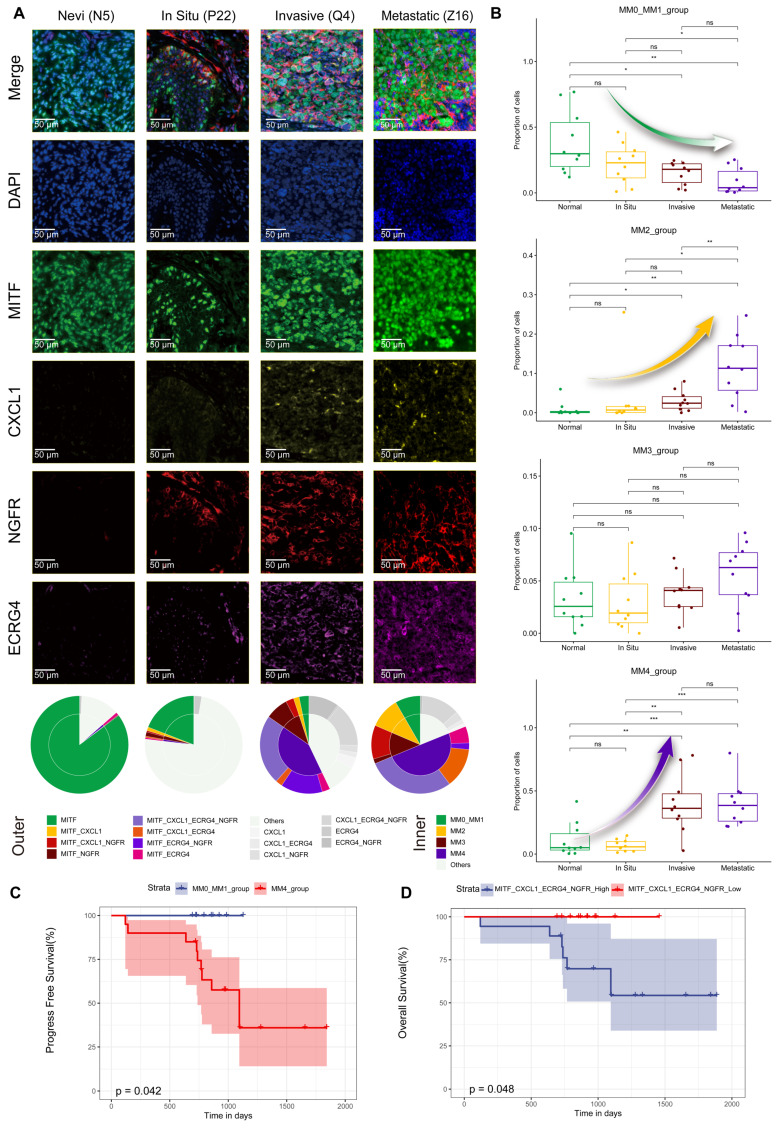
** Melanoma subgroups in relation to tumor malignancy and prognostic analysis.** A. mIHC analysis showing the distribution of MITF (green), CXCL1 (yellow), NGFR (red), and ECRG (purple) antibodies in compound nevi, *in situ* melanoma, invasive melanoma, and metastatic melanoma. 4′-6′-diamidino-2-phenylindole (DAPI; blue) indicates cell nuclei. Double-pie charts display staining results in different areas: the inner pie chart shows the melanoma-initiating cell (MM0), pigmented melanoma (MM1), intermediate transitional state melanoma (MM2), Schwann-like melanoma precursor (MM3), and Schwann cell-like melanoma (MM4) subgroups, whereas the outer ring corresponds to various antibody combinations. Specifically, cells expressing only MITF were classified into the MM0 and MM1 subgroups; those expressing MITF and CXCL1 were defined as MM2; cells expressing MITF and NGFR, regardless of CXCL1 expression, were identified as MM3; and classification as MM4 depended on the combined expression of MITF and ECRG4. Other antibody combinations were categorized as “other” types. B. Box plots comparing the proportions of MM0 and MM1, MM2, MM3, and MM4 melanoma subgroups in compound nevi, *in situ* melanoma, invasive melanoma, and metastatic melanoma. C. Comparison of PFS among the most prominent melanoma subgroups in the *in situ*, invasive, and metastatic melanoma sample cohorts. D. Kaplan-Meier survival curve analysis of melanoma subgroups expressing MITF, CXCL1, NGFR, and ECRG4.

We included melanoma samples with varying degrees of malignancy, including 10 cases each of *in situ* melanoma, invasive melanoma, and lymph node metastatic melanoma, to investigate the correlation between the malignancy level of melanoma and the distribution of its cell subtypes. In addition, 10 plantar melanocytic nevi samples were used as controls. We meticulously observed the distribution of MITF, CXCL1, NGFR, and ECRG in tissue samples using mIHC (Figure 5A and S5). The mIHC analysis revealed that in the nevi samples, MM0 and MM1 subgroups were the most prevalent, followed by those in *in situ* melanoma. Notably, a significant increase in the proportion of the MM4 subgroup was observed in the invasive and metastatic melanoma samples (Figure 5A).

We observed several significant trends after the mIHC analysis of the 40 tissue samples. Detailed clinical and pathological data are presented in Table S2. Figure 5B shows a constant decline with tumor progression in the proportions of MM0 and MM1 subgroups. Notably, the proportions of MM0 and MM1 in the compound nevus group were significantly higher than those in the invasive (P<0.05, t-test) and metastatic (P<0.01, t-test) melanoma groups. In contrast, the proportion of the MM2 subgroup significantly increased throughout tumor development (Figure 5B). This increase was particularly pronounced in metastatic melanomas, where the proportion of MM2 significantly exceeded that of the invasive (P<0.01, t-test), *in situ* (P<0.05, t-test), and compound nevus (P<0.01, t-test) groups. However, the MM3 subgroup did not exhibit significant differences in proportions across groups (P>0.05, t-test, Figure 5B). Conversely, the MM4 subgroup presented an opposite trend, with its proportion in tissue samples significantly increasing as the tumor worsened (Figure 5B). The proportion of MM4 in the metastatic group did not significantly differ from that in the invasive group (P>0.05, t-test); however, it was substantially higher than that in the *in situ* (P<0.001, t-test) and compound nevus (P<0.001, t-test) groups.

We conducted a postoperative follow-up of 30 patients who underwent surgical melanoma treatment, with a median follow-up period of 981 days. The analysis revealed that patients in the MM4 subgroup had shorter progression-free survival, with all recorded outcome events (such as recurrence, metastasis, or death) occurring within this subgroup (Figure 5C). In addition, patients expressing all four markers (MITF, CXCL1, ECRG4, and NGFR) had significantly reduced overall survival (Figure 5D).

### HMGA2 is a key transcription factor that drives the transition of the PM to a Schwann cell-like fate

**Figure 6:**
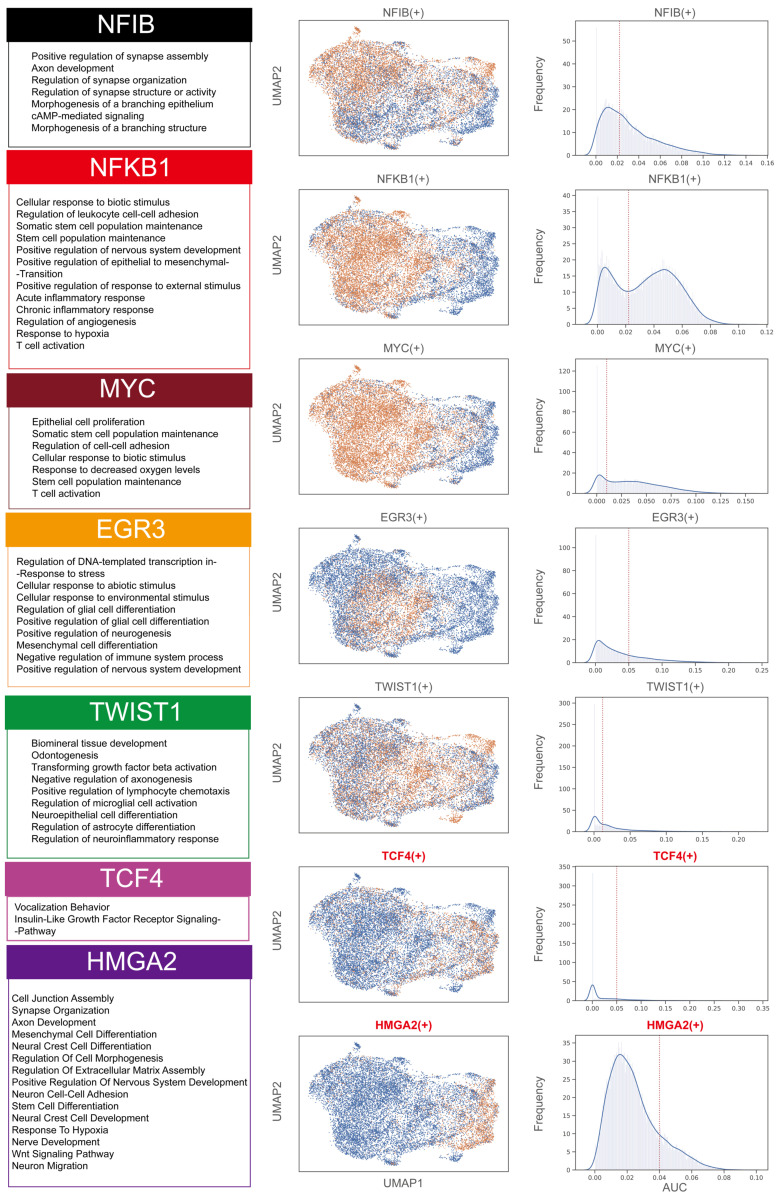
** Assessment of activity in key transcription factor regulatory networks of melanoma subgroups.** Enrichment analysis of the Gene Ontology Biological Process (GOBP) for the regulatory networks of *MYC*, *NFKB1*, *NFIB*, *EGR3*, *HMGA2*, *TCF4*, and *TWIST* transcription factors (left). UMAP representation showing the activity of these transcription factors, where yellow represents an activated state in cells (middle), and the distribution of area under the curve (AUC) values for selected transcription factor regulons (right).

A comprehensive analysis of transcription factors in melanoma cell subgroups revealed significant associations between specific transcription factors and different subgroups (Figure 6 and Figures S6). Specifically, transcription factors such as *MYC* and *NFKB1* were closely associated with the MM0, MM1, and MM2 subgroups, regulating gene networks associated with the maintenance of stem cell populations. In the MM1 subgroup, the gene network regulated by the *NFIB* transcription factor primarily involved synapse assembly and axon development. The *EGR3*-regulated gene network in the MM2 subgroup was associated with cellular response to environmental stimulus. The *TWIST*-regulated gene network in the MM2 subgroup was associated with differentiation of neuroepithelial cells and astrocytes. Notably, the Schwann cell-like fate subgroups (MM3 and MM4) were significantly associated with the transcription factors *TCF4* and *HMGA2*. The *TCF4*-regulated network was involved in the insulin-like growth factor receptor signaling pathway, while the *HMGA2*-regulated network was involved in neural crest cell differentiation.

**Figure 7:**
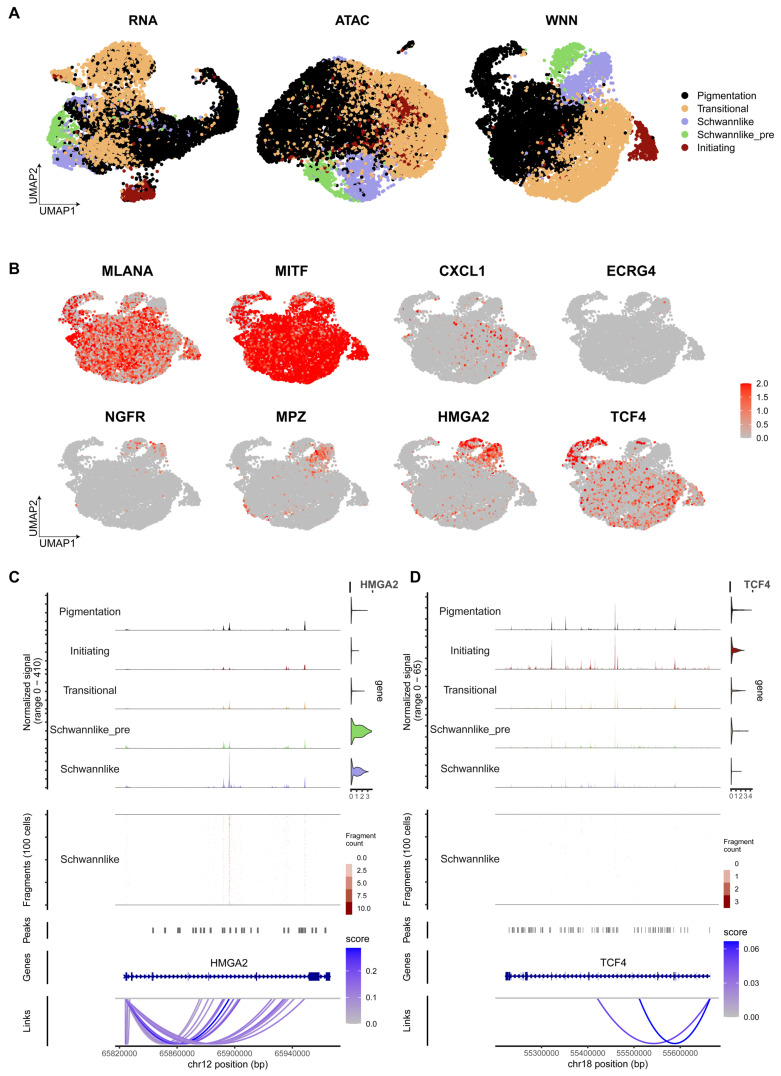
** Chromatin accessibility analysis of Schwann cell-like melanoma subgroup.** A. Integration of Single-cell Epi Multiome ATAC and Gene Expression sequencing data and subgroup annotation. The scRNA melanoma subgroup annotation (left), scATAC melanoma subgroup annotation (middle), and WNN-integrated scRNA and scATAC cell subgroup annotation (right). B. UMAP displaying the gene expression levels of *MLANA*, *MITF*, *CXCL1*, *ECRG4*, *NGFR*, *MPZ*, *HMGA2*, *TCF4*, and *SOX10*. C. Chromatin accessibility track analysis and gene expression analysis, with violin plots shown to the right of the chromatin accessibility tracks for *HMGA2* across all melanoma cell types. Gene structure is shown below the tracks. D. Chromatin accessibility track analysis and gene expression analysis, with violin plots shown to the right of the chromatin accessibility tracks for *TCF4* across all melanoma cell types. Gene structure is shown below the tracks.

To identify the key regulator driving the transition to the Schwann-like melanoma subgroup, we integrated additional single-cell Epi-Multiome ATAC and gene expression sequencing data from 5 melanoma samples. (Figure 7A-B, Table S3). Peak analysis of chromatin accessibility suggested that *HMGA2* is associated with increased chromatin accessibility and plays a critical role in shaping the chromatin landscape characteristic of Schwann-like melanoma subgroups, indicating its potential as a key regulator in their differentiation (Figure 7C-D).

**Figure 8:**
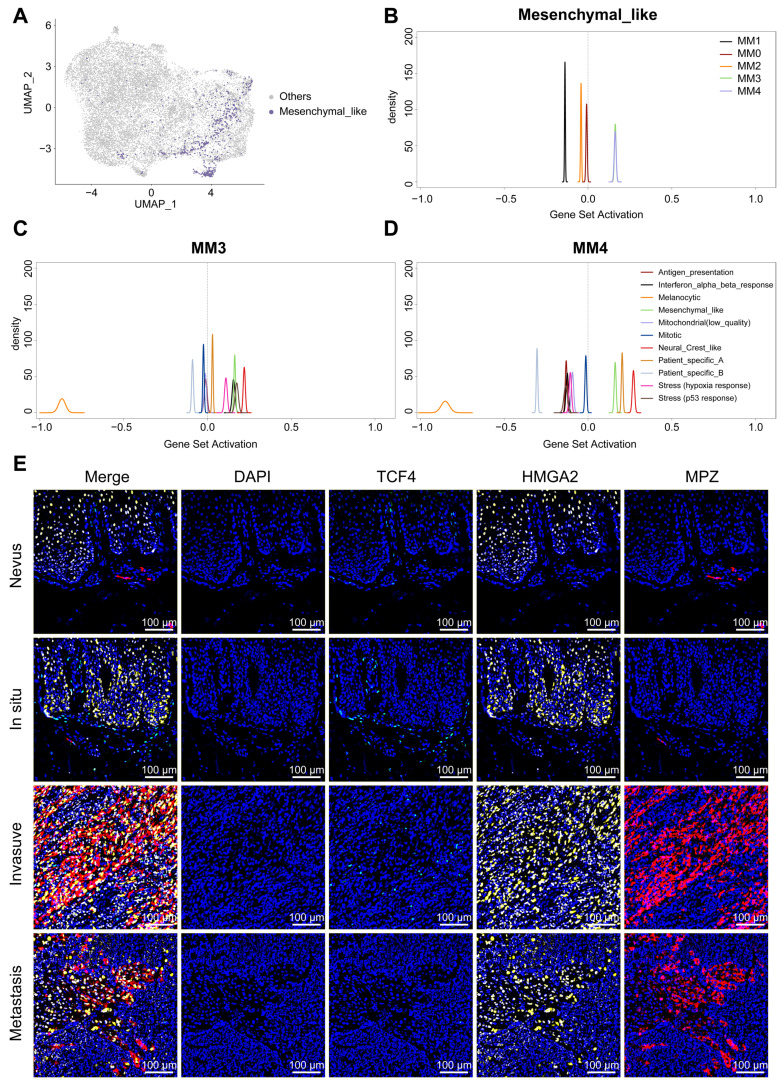
** The intersection and divergence of Schwann's fate and mesenchymal features.** A. The UMAP illustrates the mapping of mesenchymal melanoma cells, as defined by Pozniak *et al.* to the Schwann cell-like melanoma subgroups we have identified. B. Qusage analysis reveals the signaling characteristics of plantar melanoma subgroups within mesenchymal melanoma cells. C. Qusage analysis reveals the signaling characteristics of melanoma subgroups as defined by Pozniak *et al.* within the MM3 and MM4 subgroups. D. mIHC analysis showing the distribution of TCF4 (green), HMGA3 (yellow) and MPZ (red) antibodies in compound nevi, *in situ* melanoma, invasive melanoma, and metastatic melanoma. 4′-6′-diamidino-2-phenylindole (DAPI; blue) indicates cell nuclei.

To investigate the distinct roles of *TCF4* and *HMGA2* during the transition to Schwann-like melanoma identities, and to understand the relationship between the MM4 subgroup and the mesenchymal cell subgroup defined by the Pozniak *et al.* team 11, we first used Seurat for gene mapping (Figure 8A). The analysis indicated that the mesenchymal-like melanoma subgroup is included within the Schwann cell-like fate subgroups, predominantly converging at the intersection of MM3 and MM4 subgroups (Figure 8A). Quantitative Set Analysis for Gene Expression (Qusage) analysis revealed that these cells exhibited prominent MM3 and MM4 signaling characteristics (Figure 8B). However, in addition to the enhanced mesenchymal-like melanoma signals observed in our two subgroups, distinct neural crest-like features were also observed (Figure 8C and 8D). This suggests that although the MM4 subgroup has strong similarities to the mesenchymal melanoma subgroups, it still has its own distinct characteristics. We then further differentiated these subgroups within the spatial context of the tissue and examined the expression of TCF4 and HMGA2 in the MM4 subgroup across normal nevi and melanomas of varying malignancy (Figure 8E). The results showed a significant co-expression of HMGA2 and TCF4 in invasive melanomas. However, in metastatic samples, predominantly HMGA2 showed high co-expression with MPZ (Figure 8E).

### HMGA2: a driver of neural characteristics, migration, immune escape and metastasis in melanoma

**Figure 9:**
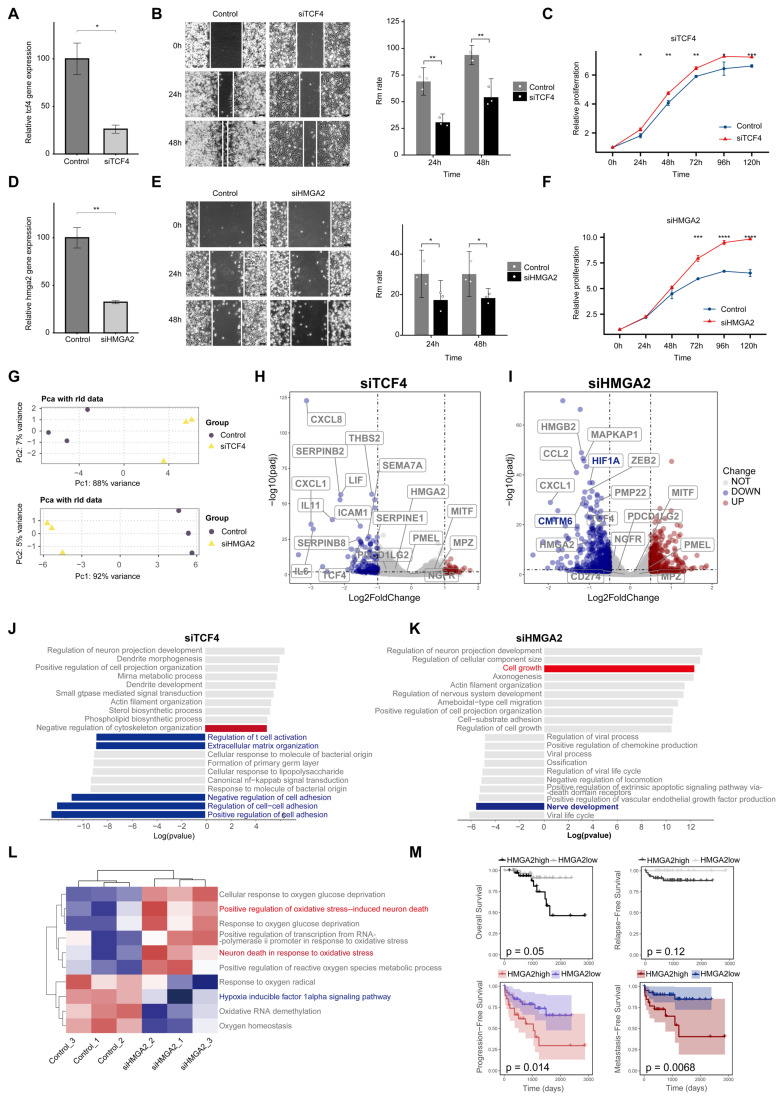
***HMGA2* drives neural development, immune evasion, and lymph node metastasis in melanoma.** A. qPCR validation of gene knockdown efficiency for siTCF4. B. Scratch assays comparing migration abilities of siTCF4 cells versus control cells. C. CCK8 Cell proliferation curves showing changes in proliferation rates of siTCF4 cells compared to control cells. D. qPCR validation of gene knockdown efficiency for siHMGA2. E. CCK8 Cell proliferation curves showing changes in proliferation rates of siHMGA2 cells compared to control cells. F. Scratch assays comparing migration abilities of siHMGA2 cells versus control cells. G. PCA analysis revealing key differences between melanoma cells after siTCF4 (top) and siHMGA2 (down) treatments versus controls. H. Volcano plots displaying genes with significantly altered expression levels in siTCF4 cells relative to controls. Red points indicate significantly upregulated genes, blue points indicate significantly downregulated genes, and gray points indicate genes with no significant changes. Selection criteria: adjusted P-value (P.adjust) < 0.05 and absolute log2 fold change (log2FC) > 0.5. Genes of interest are highlighted in boxes. I. Volcano plots showing genes with significantly altered expression levels in siHMGA2 cells compared to controls (Screening criteria are the same as in H). J. GOBP analysis showing the top 10 upregulated and downregulated gene pathways in siTCF4 cells compared to controls. Pathways of interest are color-coded: red indicates enhanced pathway activity, and blue indicates suppressed pathway activity. The x-axis displays the log(P-value) of pathway genes, with positive values indicating upregulation and negative values indicating downregulation. K. GOBP analysis showing the top 10 upregulated and downregulated gene pathways in siHMGA2 cells compared to controls (Screening criteria are the same as in H). L. GSVA analysis quantifying differences in oxygen-related pathway activities between siHMGA2 and control groups, with red indicating enhanced pathway activity and blue indicating suppressed activity. M. Kaplan-Meier survival curves comparing overall survival (OS), progression-free survival (PFS), metastasis-free survival (MFS), and recurrence-free survival (RFS) between patients with high and low HMGA2 expression levels.

To investigate the role of *TCF4* and *HMGA2* in PM, we performed functional experiments using the PM cell line LM-MEL-45. After silencing *TCF4* and *HMGA2* with siRNA, we observed a significant decrease in the migration ability of melanoma cells, accompanied by a marked increase in proliferation capacity (Figure 9A-F). We then performed bulk RNA sequencing to further investigate the functional impact of these gene knockdowns (Figures 9G-L). Analysis revealed that *CXCL2*, a marker of transitional states, was significantly downregulated and a cross-regulatory relationship was observed between *TCF4* and *HMGA2* (Figures 9H and 9I).

Gene enrichment analysis of the DEG revealed that *TCF4* knockdown primarily led to a significant reduction in extracellular matrix organization and cell adhesion capacity, along with suppressed immune functions (Figure 9J). Notably, *HMGA2* knockdown resulted in a significant reduction in neural development-related functions (Figure 9K). In addition, we observed a significant downregulation of the hypoxia inducible factor HIF1A (Figure 9I). GSVA-GOBP analysis showed that *HMGA2* knockdown reduced the tolerance of melanoma cells to hypoxic conditions, potentially inducing the death of neurotype melanoma cells (Figure 9L). Among the expression of immune-related genes, we observed a significant downregulation of *CMTM6*, a core stabiliser of *PD-L1* 33. This change may weaken the ability of tumor cells to evade the immune system.

Finally, we performed bulk RNA sequencing on 68 melanoma patients and analysed clinical correlations using follow-up data. The results showed that patients with high *HMGA2* expression had a worse prognosis, characterised by higher rates of PFS and MFS (Figure 9M).

## Discussion

PM is a subtype of melanoma characterized by poorer prognosis and less effective immunotherapy outcomes, posing a challenge in clinical management due to its heterogeneity. Using single-cell multi-omics sequencing technology, we identified a novel melanoma subgroup in PM samples and uncovered its unique evolutionary trajectory, which may explain the underlying reasons for these phenomena. This subgroup not only displays functions associated with mesenchymal cells but also possesses a unique ability to sense external stimuli (Figure 2C). We also observed a reduction in pigment production capabilities (as indicated by the decreased expression of *MLANA*, *MITF*, *TYR*, and *PMEL*) and an increase in neural-related functions (as indicated by the increased expression of *NGFR* and *MPZ*) within this subgroup (Figure S2). Tissue cell type scoring revealed a strong association of identity with Schwann cells. By tracing the origin of this cell population, we discovered that it is derived from a melanoma cell subgroup with the strongest stemness potential. This subgroup has the capacity to differentiate towards a pigment-producing melanoma cell or, through an intermediate stage, unlocking the potential for differentiation towards Schwann cells, ultimately evolving into *MPZ*-positive melanoma subgroup. Based on these considerations, we have redefined this novel subgroup as the Schwann-like melanoma cell subgroup.

This phenomenon is related to the origin of melanocytes from neural crest cells, specifically the ability of neural crest cells to differentiate into different cell types during development 34. Typically, after neural tube closure during embryonic development, neural crest cells migrate to the skin along two different pathways to complement melanocytes 34, 35. One pathway is the dorsolateral migration pathway between the dermomyotome of the somites and the overlying ectodermal epithelium 34. The other is the ventromedial pathway between the neural tube and sclerotome of the somites 34, 35. During the latter migration, these neural crest cells gradually transform into Schwann cell precursors, giving them the pluripotency to differentiate into melanocytes or Schwann cells 34, 35. Subsequently, Schwann cell progenitors differentiate into Schwann cells as they migrate along the nerve axis, while cells that are separated from the nerves tend to become melanocytes 34, 35. This pathway is more closely associated with the production of plantar melanocytes 35. Our research shows that the progression of melanoma disrupts the differentiation pathways of normal melanocytes, leading them to a mixed state that has both pigmentary functions and neural properties. In this state, the cells are unable to fully mature into normal melanocytes or differentiate into typical Schwann cells. This does not represent a complete regression to the neural crest cell state, as it remains in an actively differentiating state. During this process, some genes from the embryonic development stage may be reactivated 36. Kastriti 36 *et al.* found that specific tumor subgroups in uveal melanomas correspond to different developmental stages of Schwann precursor/neural glial cells.

What does the differentiation of melanoma towards Schwann cells entail? The first characteristic is that tumor cells can undergo immune escape. On the one hand, Pozniak 11 *et al.* revealed that *TCF4*, primarily expressed in mesenchymal-like melanoma cells, inhibits melanin synthesis, antigen presentation, and the transcriptional program of the interferon signaling pathway, which may directly promote immune cell escape and/or resistance to immunotherapy. We found that the characteristics of this mesenchymal melanoma are contained within the MM3 and MM4 subgroups in terms of gene expression and functional characteristics. On the other hand, in our study, cellular communication analysis revealed that the MM4 subtype had the highest volume of communication data among all tumor cell subtypes and was also the subtype with the most interactions with immune cell types. The MM4 subtype communicates with various immune cell types through the pairing of LAMC1-CD44. LAMC1, as a component of laminin, can bind to CD44 37. Laminin also supports and influences the migration and function of immune cells 37. CD44 is prominently expressed on lymphocytes, smooth muscle cells, fibroblasts, and various epithelial cells and is involved in lymphocyte homing, cell adhesion and aggregation, cell migration, leukocyte activation, lymphocyte generation, maintenance of memory T cells, and tumor progression and metastasis 38, 39. Blocking CD44 can result in reduced lymphocyte and leukocyte infiltration 39. Additionally, In the single-cell spatial transcriptomics data, we observed increased scoring of exhausted T cells surrounding the MM4 subgroup. Finally, we identified two critical transcription factors, TCF4 and HMGA2, that are closely associated with the transition to a Schwann-like melanoma fate (Figure 6). When TCF4 was knocked down in cell lines using siRNA technology, a marked impairment of immune function was observed. In particular, knockdown of *HMGA2* led to a significant reduction in the expression of *CMTM6*, a key stabilizer of *PD-L1* 33. *CMTM6* plays a critical role in tumor immune evasion by maintaining the stability and function of *PD-L1* 33. When *CMTM6* expression is reduced, both the expression and activity of *PD-L1* are significantly reduced, impairing the ability of tumor cells to suppress the immune system and weakening their immune evasion ability. This finding suggests that *HMGA2* may be a promising new immunotherapeutic target for melanoma patients who are resistant to immunotherapy.

The second characteristic is the increased invasiveness of tumor cells. We conducted an mIHC analysis of samples from patients with melanoma to understand the clinical significance of this discovery. We replicated the distribution of different melanoma subgroups in the tissue samples by identifying specific markers. Notably, significant subgroup differences in spatial distribution were observed in a sample from a patient with invasive melanoma. As tumor invasion intensified, the proportion of MM4 cells increased (Figure 4). These findings suggest a close link between the trend of melanoma differentiation toward Schwann-like characteristics and its invasive capacity. To further validate this finding, we expanded the sample size for mIHC analysis to include patients with varying degrees of melanoma malignancy and collected prognostic data. Immunoscoring results showed that with tumor progression, the proportions of MM0 and MM1 decreased, whereas those of MM2, MM3, and MM4 increased. The MM4 subgroup was significantly associated with a shorter PFS, and patients with a high proportion of cells co-expressing MITF, CXCL1, NGFR, or ECRG4 had worse OS. This finding introduces a critical perspective in clinical and cancer biology: the need for heightened attention to the classification, quantity, and proportion of neural-type melanoma cell subgroups within tumors. Notably, an increased proportion of the MM4 subgroup is a crucial biological indicator of tumor worsening and enhanced invasiveness. This provides a new direction for future research and the development of targeted therapies for specific cell subgroups.

Growing research suggests that reduced pigment capacity in CM is associated with increased tumor aggressiveness and drug resistance 11, 40-43. Considering the biological characteristics of melanoma, it has been assumed that two main melanoma states, proliferation and invasion 44-46, are closely associated with *MITF* expression level. Malignant cells exhibit a continuum between high and low *MITF* expression, and this dynamic gene expression pattern demonstrates the plasticity and adaptability of melanoma cells at the genetic level, enabling tumor cells to transition between different states 47. Low expression of *MITF* in melanoma is usually associated with tumor invasiveness and drug resistance, potentially activating cell adhesion and extracellular matrix pathways 42, 48. Notably, various cellular stress factors, such as hypoxia, low glucose levels, amino acid limitation, and inflammatory signaling, can reduce *MITF* expression and thereby enhance melanoma cell invasiveness 45. Travnickova 42 *et al.* used zebrafish models to discover that melanomas with low levels of *MITF* exhibited significant invasiveness and treatment resistance. Melanoma cell subgroups with low MITF expression may already exist in primary tumors 48, 49 and are more prominently expressed in minimal residual lesions 41, displaying unique gene expression patterns associated with invasiveness and neural crest stem cell (NCSC) traits 41. Rambow 41 *et al.* found that drug exposure promotes the transcriptional reprogramming of other melanoma cell types towards the NCSC subgroup, increasing its proportion and becoming a major driving factor for disease recurrence and treatment resistance. This could explain why some atypical melanomas lack pigment expression and are associated with a poorer prognosis.

In our study, single-cell multi-omics analysis revealed that *HMGA2* exhibits significant chromatin accessibility in the Schwann-like melanoma subgroup, identifying it as a key transcription factor driving melanoma differentiation towards a Schwann-like fate. Notably, we detected a higher abundance of *HMGA2*-positive MM4 melanoma cells in tissue samples from patients with lymph node metastases (Figure 8E). Prognostic analysis of bulk RNA-seq data from 68 patients showed that those with high *HMGA2* expression were more likely to experience disease progression, particularly in the context of lymph node metastasis (Figure 9M). Targeting *HMGA2* may be an important strategy to prevent melanoma metastasis. Suppression of *HMGA2* expression significantly reduced the neural development capacity of melanoma cells and inhibited their ability to migrate. Importantly, we observed a marked decrease in hypoxia-inducible factor 1 alpha (*HIF1A*) expression following *HMGA2* inhibition, together with reduced activity of the hypoxia inducible factor 1alpha signaling pathway (Figure 9I and 9L). At the same time, signaling associated with neuronal death in response to oxidative stress was significantly enhanced (Figure 9L). These findings highlight the central role of *HMGA2* in melanoma progression and provide important insights for the development of novel therapeutic strategies.

This study has some limitations. First, the PM samples were obtained exclusively from the Chinese population. Therefore, future studies should expand the sample size and geographical origins to ascertain whether these findings represent the general characteristics of PM and are applicable across different ethnicities. Second, in the immunohistochemistry experiments, we grouped the MM0 and MM1 subgroups for analysis, but we failed to distinguish between the two. The current differential gene analysis did not reveal specific genes that completely differentiated the two subgroups. Lower levels of genes differentially expressed in the MM0 subgroup were also present in the MM1 subgroup. However, analyses of cell identity, stem cell properties, and cell proliferation rates showed significant differences between both groups, suggesting that MM0 represents an earlier stage than MM1 does. Finally, while gene knockdown experiments in cell lines have provided functional insights into the role of HMGA2, further validation in *in vivo* models is required to understand their relevance in melanoma progression and to explore potential therapeutic applications targeting these factors.

In conclusion, this study reveals the unique evolutionary trajectory of PM, which diverges from the pigment-producing fate and progressively differentiates towards a Schwann-like fate. During this process, PM exhibits a gradual decline in pigment function, a significant increase in immune tolerance, and an increased propensity for distant invasion and lymph node metastasis. This phenotypic transition explains why PM has a worse prognosis and lower efficacy of immunotherapy compared to melanoma in other skin sites. Functional validation further demonstrated that *HMGA2* plays a pivotal role in Schwann-like fate differentiation. Knockdown of *HMGA2* not only significantly reduced melanoma cell migration but also weakened their neural developmental potential and tolerance to hypoxic conditions. In the future, in-depth exploration of the functional mechanisms and therapeutic potential of *HMGA2* as a target may serve as a critical breakthrough to improve the treatment outcomes and prognosis of PM.

## Supplementary Material

Supplementary figures and tables.

## Abbreviations

mIHC: multiplex immunohistochemistry
HMGA2: high mobility group AT-hook 2
scRNA-seq: single-cell RNA sequencing
TCF4: transcription factor 4
MITF: microphthalmia-associated transcription factor
ECRG4: esophageal cancer related gene 4
MPZ: myelin protein zero
HMGA2: high mobility group AT-hook 2
MLANA: melanoma-associated antigen
TYR: tyrosinase
PMEL: premelanosome protein
CopyKAT: Copy Number Karyotyping of Aneuploid Tumors
SEMA3: semaphorin 3
NRXN: neurexin
PDGFR: platelet-derived growth factor receptor
NCAM: neural cell adhesion molecule
CSPG4: chondroitin sulfate proteoglycan 4
LAMC1: Laminin subunit gamma 1
MPZL1: myelin protein zero Like 1
GSEA-GOBP: Gene Set Enrichment Analysis-Gene Ontology Biological Process
CytoTRACE: Cellular Trajectory Reconstruction Analysis using Gene Counts and Expression
WebCSEA: Web-based cell-type-specific enrichment analysis
MHC-II: major histocompatibility complex class II
GSVA-GOBP: Gene Set Variation Analysis-Gene Ontology Biological Process
NGFR: nerve growth factor receptor
CXCL1: C-X-C motif chemokine ligand 1
PFS: progression-free survival
MYC: MYC proto-oncogene, BHLH transcription factor
NFKB1: nuclear factor kappa B subunit 1
NFIB: nuclear factor I B
EGR3: early growth response 3
TWIST: twist family bHLH transcription factor 1
ATAC: Assay for transposase-accessible chromatin
Qusage: Quantitative set analysis for gene expression
CMTM6: CKLF-like MARVEL transmembrane domain containing 6
PD-L1: programmed death-ligand 1
MFS: metastasis-free survival
HIF1A: hypoxia-inducible factor 1-alpha
NCSC: neural crest stem cells
siRNA: small interfering RNA
DEG: differentially expressed genes
OS: overall survival
TME: tumor microenvironment
MM: melanoma cells
KCs: keratinocytes
Fbs: fibroblasts
ECs: endothelial cells
Bcells: B cells
Ap_KCs: epidermal appendages keratinocytes
SWs: Schwann cells
Ly_ECs: lymphatic endothelial cells
Mast: mast cells
Mo&Mac: monocytes and macrophages
Neus: neurons
PCs: pericytes
Tcells: T cells
CD4: CD4+ T cells
Treg: Regulatory T cells
CD4_LHFPL6: CD4+ LHFPL6+ T cells
CD8: CD8+ T cells
Tex: Exhausted T cells
NK1, NK2: Natural killer cell subsets
cDC1, cDC2, cDC3: Dendritic cell subsets
LC: Langerhans cells
MacI, MacII: Macrophage subsets
Mast: Mast cells
Neus: Neutrophils
