# Supplementary material for: A distinct subgroup with Schwann-like differentiation presents immune resistance and poor prognosis in plantar melanoma
Source: Theranostics. 2025 Mar 3;15(9):3900–23. doi: 10.7150/thno.99526 (PMC11980676; doi:10.7150/thno.99526)
Supplement: Supplementary file 1 — Supplementary figures and tables. [file thnov15p3900s1.pdf]

Supplemental Information

Supplementary Table 1: Clinical characteristics of single-cell sequencing samples

Sample	Sex	Age (years)	Position	Breslow thickness (mm)	Diagnosis
MM13	F	49	Heel	0	In Situ
MM25	F	59	Big toe	0	In Situ
MM28	M	76	Heel	0	In Situ
MM47	M	37	Heel	0	In Situ
MM49	M	70	Plantar	0	In Situ
MM14	F	41	Heel	1.575	Invasive
MM2	M	76	Plantar	3.15	Invasive
MM21	F	30	Big toe	2.17	Invasive
MM24	F	78	Plantar	2	Invasive
MM27	F	58	Big toe	1.36	Invasive
MM29	F	75	Heel	4	Invasive
MM30	M	82	Plantar	1.75	Invasive
MM33	M	62	Plantar	6.73	Invasive
MM35	F	48	Plantar	3.75	Invasive
MM38	F	58	Heel	5	Invasive
MM40	M	64	Heel	4	Invasive
MM48	M	70	Heel	11	Invasive
MM50	F	58	Plantar	5.32	Invasive
MM6	F	69	Plantar	5.85	Invasive
MM7	F	37	Heel	2.75	Invasive
NP2	F	12	Plantar	N/A	normal
NP6	F	27	Plantar	N/A	normal
NP7	F	49	Plantar	N/A	normal
NP9	F	42	Plantar	N/A	normal
NP13	F	39	Plantar	N/A	normal
NP15	F	39	Plantar	N/A	normal

N/A, Not applicable; M, Male; F, Female.

Supplementary Table 2: Clinical characteristics of the specimens used for multicolor immunohistochemistry

Sample	Sex	Age (years)	Position	Diagnosis
N1	M	54	Plantar	Compound Nevus
N10	F	44	Plantar	Compound Nevus
N2	F	53	Heel	Compound Nevus
N3	M	43	Plantar	Compound Nevus
N4	M	80	Plantar	Compound Nevus
N5	M	49	Plantar	Compound Nevus
N6	M	43	Heel	Compound Nevus
N7	F	61	Plantar	Compound Nevus
N8	F	57	Toes	Compound Nevus
N9	F	58	Plantar	Compound Nevus
P21	F	23	Plantar	In Situ
P22	F	51	Plantar	In Situ
P23	F	62	Heel	In Situ
P24	F	51	Toes	In Situ
P25	F	55	Heel	In Situ
P26	F	55	Plantar	In Situ
P27	M	38	Toes	In Situ
P28	F	37	Plantar	In Situ
P29	F	50	Toes	In Situ
P30	F	55	Plantar	In Situ
Q1	M	56	Heel	Invasive
Q10	M	93	Heel	Invasive
Q2	M	85	Heel	Invasive
Q3	F	66	Toes	Invasive
Q4	F	67	Plantar	Invasive
Q5	F	74	Plantar	Invasive
Q6	F	69	Heel	Invasive
Q7	F	70	Toes	Invasive
Q8	F	74	Heel	Invasive

Q9	M	64	Heel	Invasive
Z11	M	86	Inguinal lymph nodes	Metastatic
Z12	M	65	Inguinal lymph nodes	Metastatic
Z13	M	62	Inguinal lymph nodes	Metastatic
Z14	M	65	Inguinal lymph nodes	Metastatic
Z15	F	70	Inguinal lymph nodes	Metastatic
Z16	F	68	Inguinal lymph nodes	Metastatic
Z17	F	65	Inguinal lymph nodes	Metastatic
Z18	M	52	Popliteal lymph nodes	Metastatic
Z19	M	62	Inguinal lymph nodes	Metastatic
Z20	F	64	Inguinal lymph nodes	Metastatic

M, Male; F, Female.

Supplementary Table 3: Clinical characteristics of the specimens used for Chromium Single Cell Multiome ATAC + Gene Expression Sequencing

**Sample**	**Sex**	**Age (years)**	**Position**	**Diagnosis**
MM4	M	52	Plantar	Invasive
MM26	M	58	Heel	Invasive
MM58	F	50	Heel	Invasive
MM59	M	71	Plantar	Invasive
MM60	M	50	Plantar	Invasive

M, Male; F, Female.

Supplementary Table 4: Sequences of RT-qPCR primers

Gene	Forward/Reverse	Sequence 5'-3'	Species
GAPDH	Forward	GACTCATGACCACAGTCCATGC	Human
GAPDH	Reverse	AGAGGCAGGGATGATGTTCTG	Human
TCF4	Forward	CAAGCACTGCCGACTACAATA	Human
TCF4	Reverse	CCAGGCTGATTCATCCCACTG	Human
HMGA2	Forward	ACCCAGGGGAAGACCCAAA	Human
HMGA2	Reverse	CCTCTTGGCCGTTTTTCTCCA	Human

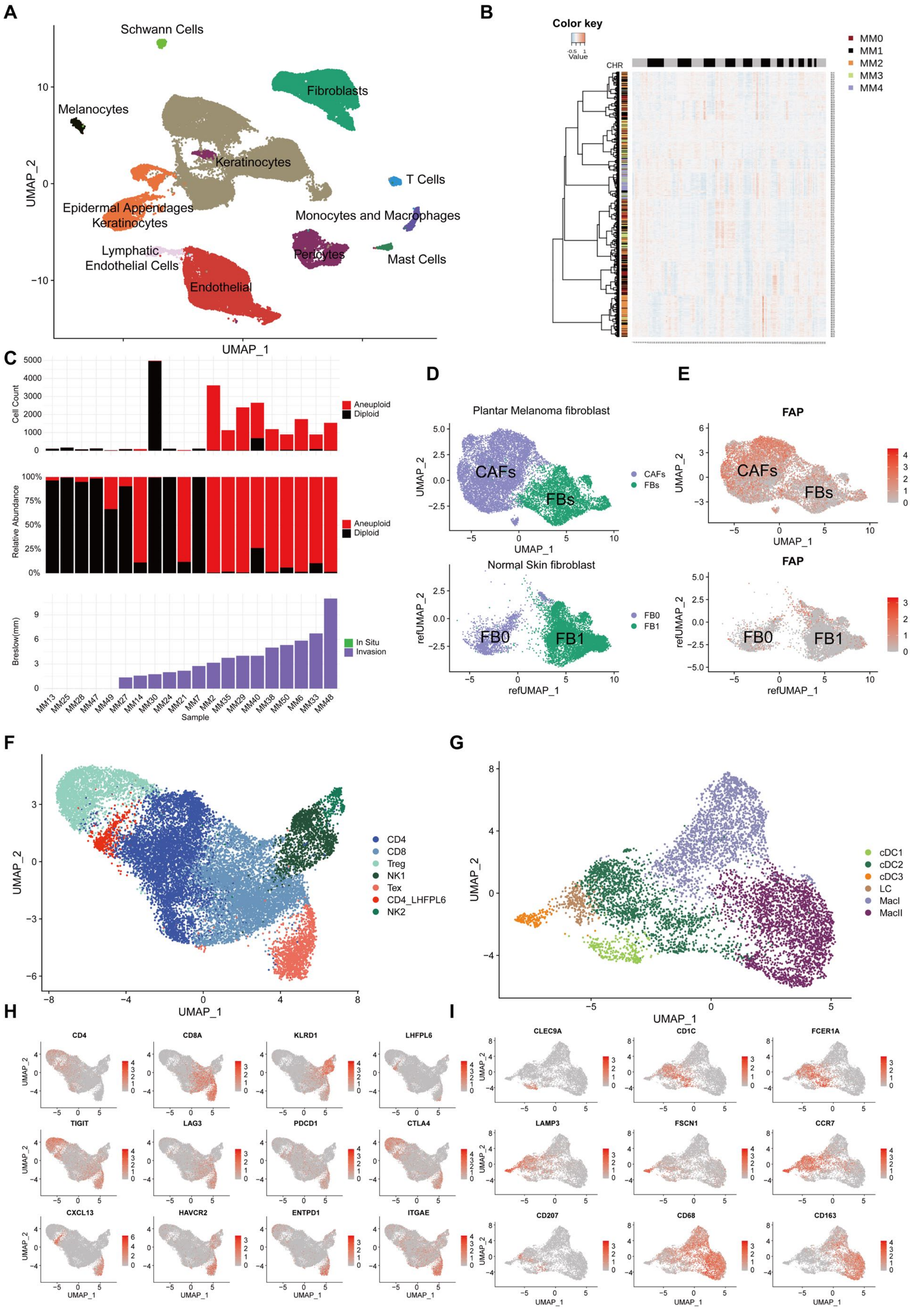

Supplementary Figure 1. Results of single-cell sequencing analysis in plantar melanoma samples

A. Uniform manifold approximation and projection (UMAP) displays the main cell types in normal plantar skin tissue.

B. The heatmap shows Copykat's predictions of copy number variations. Rows represent individual cells, whereas columns are 220 kb bins in genomic order.

C. Bar charts show the number of melanoma cells in each sample (top); stacked proportion charts display the copy number variations in melanoma predicted by Copykat in each patient (middle); bar charts show the Breslow invasion depth of tumors in each sample (bottom). Red represents aneuploidy, black represents diploidy, green represents in situ melanoma, and purple represents invasive melanoma.

D. UMAP illustrates the mapping of fibroblasts from normal tissues to the tumor fibroblast subgroups.

E. UMAP illustrates the expression of cancer-associated fibroblast markers (FAP) in fibroblast subsets of melanoma (top) and normal tissues (down).

F. UMAP shows the distribution of T cell subtypes in melanoma.

G. UMAP illustrates the distribution of monocyte and macrophage cell subtypes in melanoma.

H. UMAP shows the distribution and expression of T cell markers (*CD4*, *CD8A*), NK T cell markers (*KLRD1*), LHFPL6+ Tcell markers (*LHFPL6*), exhausted T cell markers (*TIGIT*, *LAG3*, *PDCD1*, *CTLA4*, *CXCL13*, and *HAVCR2*) and T cell dysfunction markers (*ENTPD1* and *ITGAE*). CD4: CD4+ T cells; Treg: Regulatory T cells; CD4_LHFPL6: CD4+ LHFPL6+ T cells; CD8: CD8+ T cells; Tex: Exhausted T cells; NK1, NK2: Natural killer cells subsets.

I. UMAP shows the distribution and expression of specific identity markers of DC1 subsets (*CLEC9A*), DC2 subsets (*CD1C*, *FCER1A*), DC3 subsets (*LAMP3*, *FSCN1*, *CCR7*), Langerhans cells (*CD207*), Macrophage I subsets (*CD68*), Macrophage I subsets (*CD163*). cDC1, cDC2, cDC3: Dendritic cells subsets; LC: Langerhans cells; MacI, MacII: Macrophage subsets; Mast: Mast cells; Neus: Neutrophils.

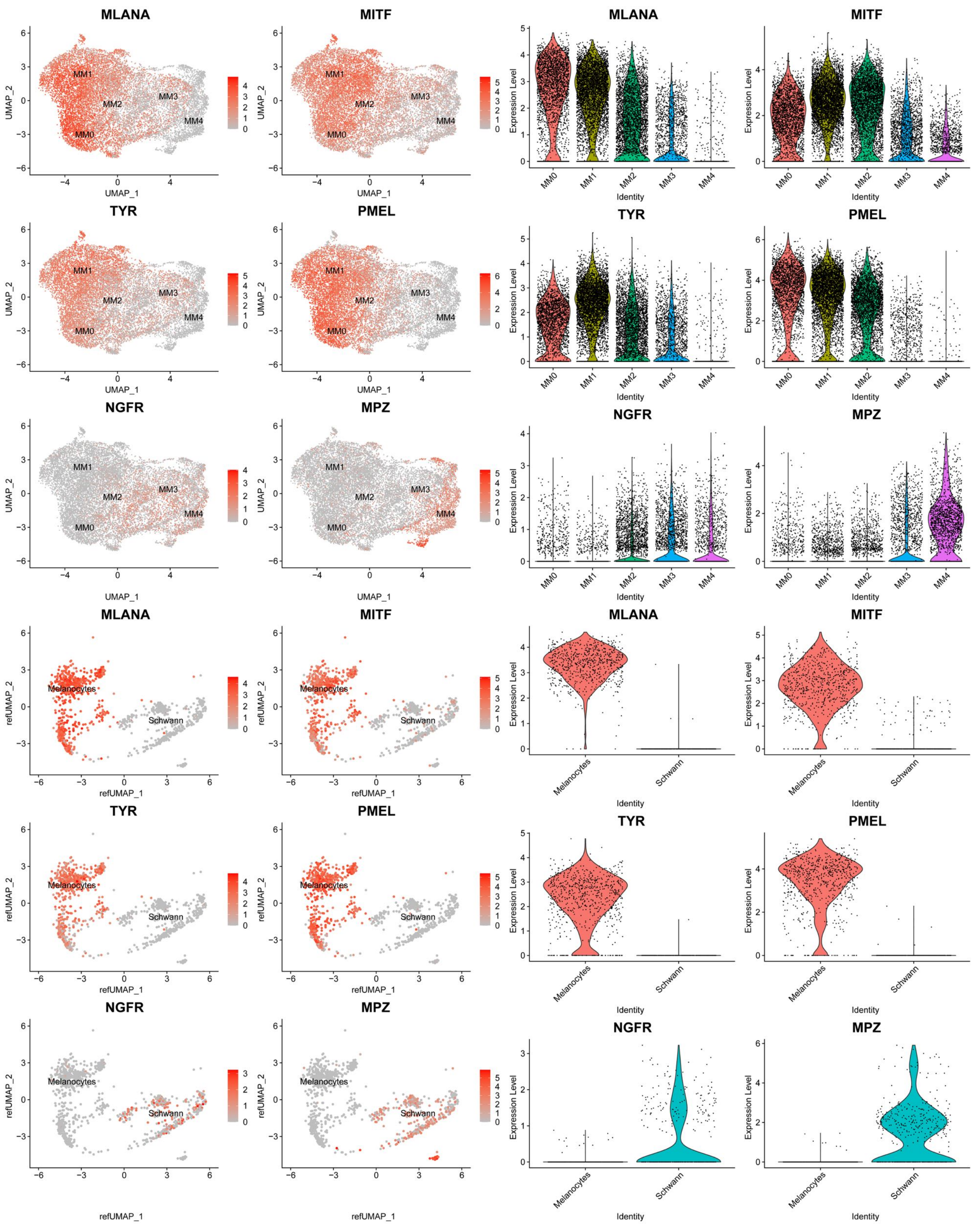

Supplementary Figure 2. Melanoma cells can simultaneously express characteristic markers associated with melanocytes and neurons

The uniform manifold approximation and projection (UMAP) plot illustrates the distribution of markers associated with pigment (*MLANA*, *MITF*, *TYR*, and *PMEL*) and neural (*NGFR* and *MPZ*) functions across melanoma subtypes, melanocytes, and Schwann cells (left). Meanwhile, the violin plot shows the specific expression levels of these pigments and neural function-related markers in the aforementioned cell types (right).

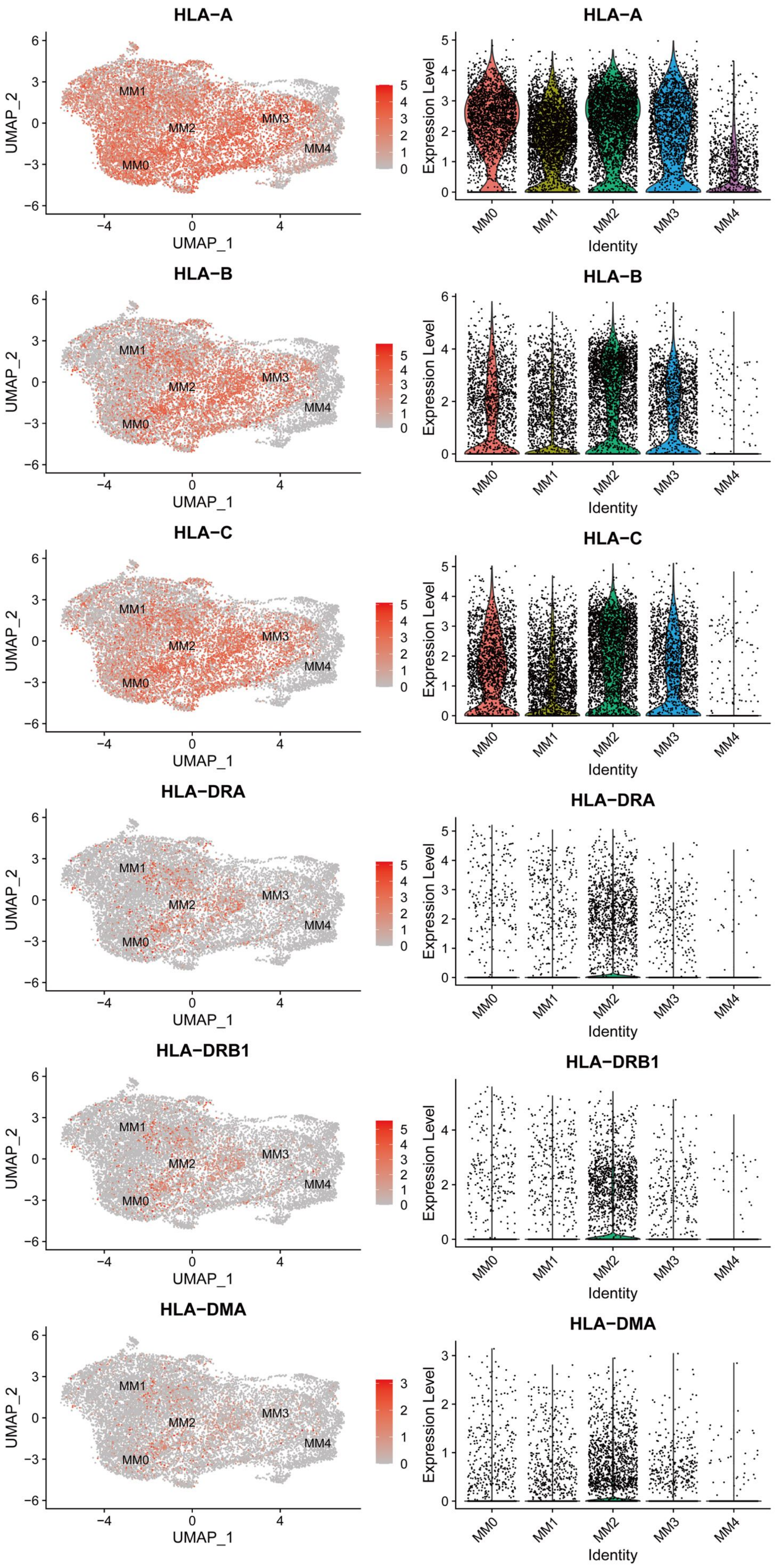
HLA−A
HLA−B
HLA−C
HLA−DRA
HLA−DRB1
HLA−DMA
UMAP_1
UMAP_2
MM0
MM1
MM2
MM3
MM4
Expression Level
Identity

Supplementary Figure 3. Expression of major histocompatibility complexes (MHCs) in melanoma subtypes

The uniform manifold approximation and projection (UMAP) plot illustrates the expression of MHC class I (*HLA-A*, *HLA-B*, and *HLA-C*) and class II complexes (*HLA-DRA*, *HLA-DRB1*, and *HLA-DMA*) in melanoma subtypes (left panel). The violin plot illustrates the expression levels of these markers in melanoma subtypes (right panel).

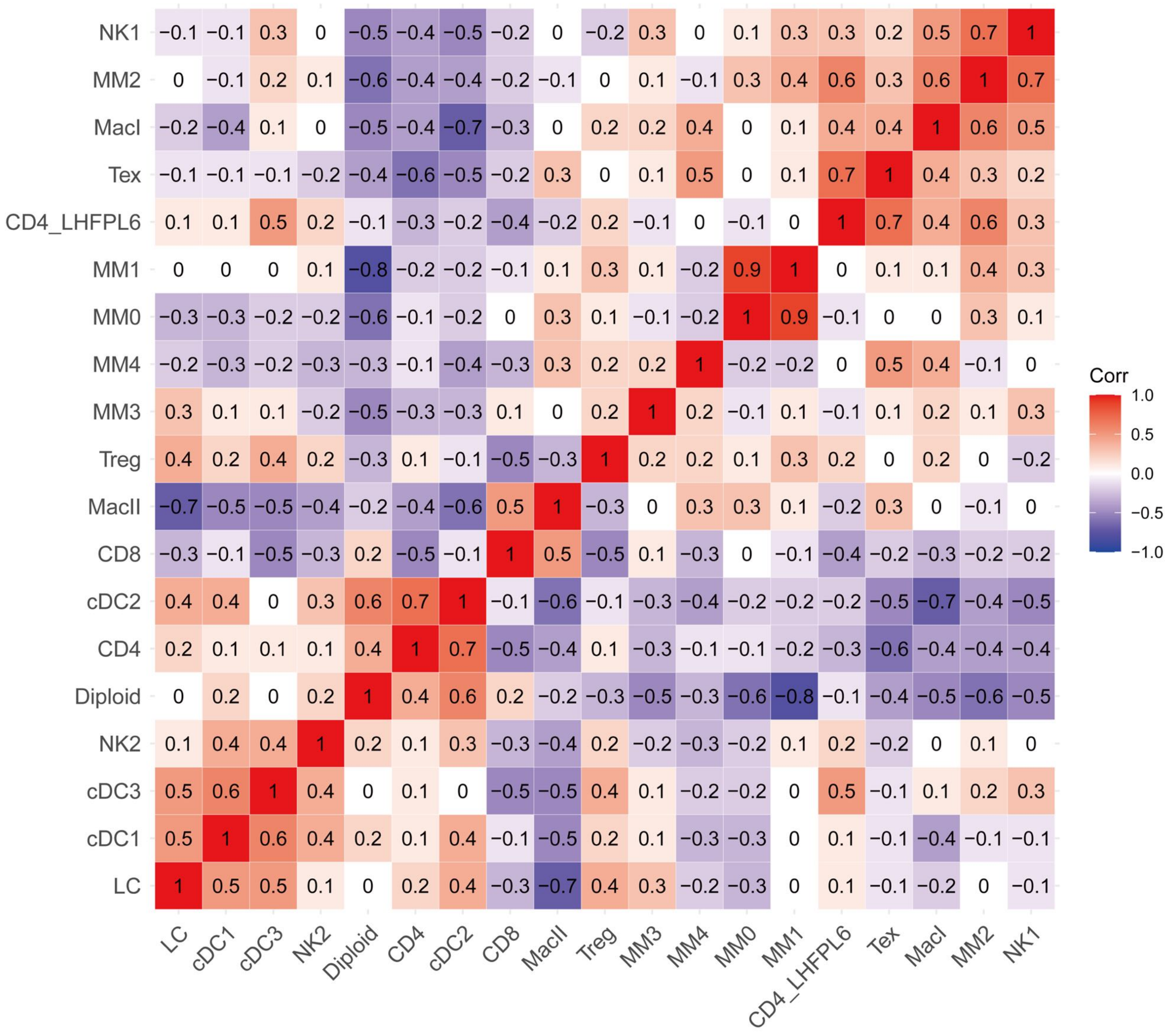

Supplementary Figure 4. Correlation analysis between MM4 abundance and immune cell subpopulations in melanoma

This heatmap illustrates the correlation coefficients between different melanoma subpopulations (MM0-MM4) and different immune cell types, including T cells (Tex, Treg, CD4, CD8), macrophages (MacI, MacII), dendritic cells (cDC1, cDC2, cDC3) and natural killer (NK) cells. The colour scale represents the strength of the correlation, with red indicating a positive correlation and blue indicating a negative correlation.

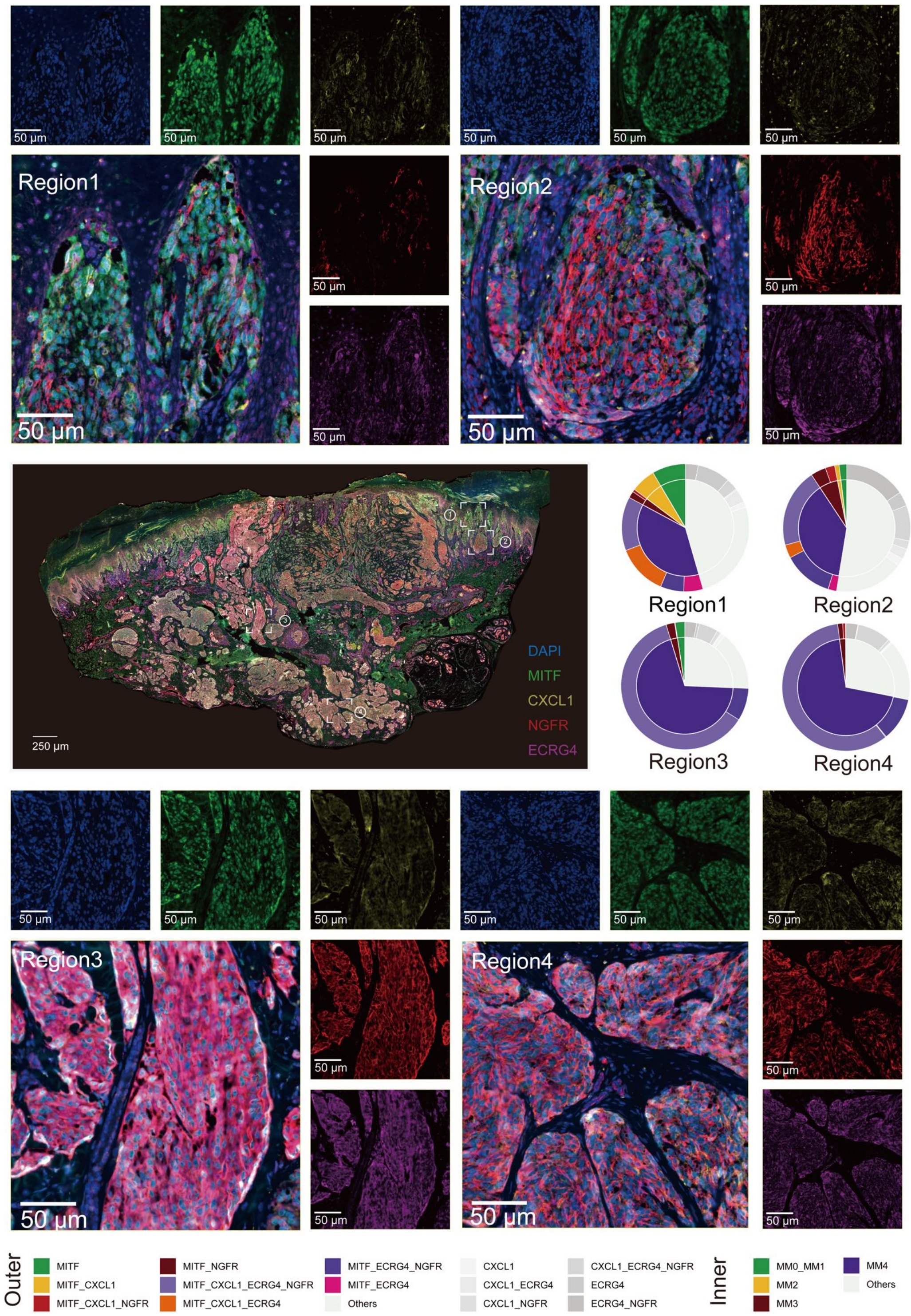

Supplementary Figure 5. Multiple immunohistochemistry spatial distribution of melanoma subgroups in invasive melanoma tissue

We selected a case of invasive melanoma with a Breslow thickness of 5.5 mm. Four different areas from the epidermis to the dermis were selected to demonstrate the distribution of MITF (green), CXCL1 (yellow), NGFR (red), and ECRG (purple) antibodies using mIHC. 4′-6′-diamidino-2-phenylindole (DAPI; blue)

indicates the cell nuclei. A double pie chart displays the staining results in different areas; the inner pie chart shows the melanoma-initiating cell (MM0), pigmented melanoma (MM1), intermediate transitional state melanoma (MM2), neuron-like melanoma (MM3), and Schwann cell-like melanoma (MM4) subgroups, whereas the outer ring corresponds to different antibody combinations. Specifically, cells expressing only the transcription factor MITF were classified into the MM0 and MM1 subgroups; cells expressing MITF and CXCL1 were defined as the MM2 subgroup; and cells expressing MITF and NGFR, regardless of whether they expressed CXCL1 as well, were identified as the MM3 subgroup. The MM4 subgroup was determined based on the combined expression patterns of MITF and ECRG4. Other antibody combinations were categorized as "other" types.

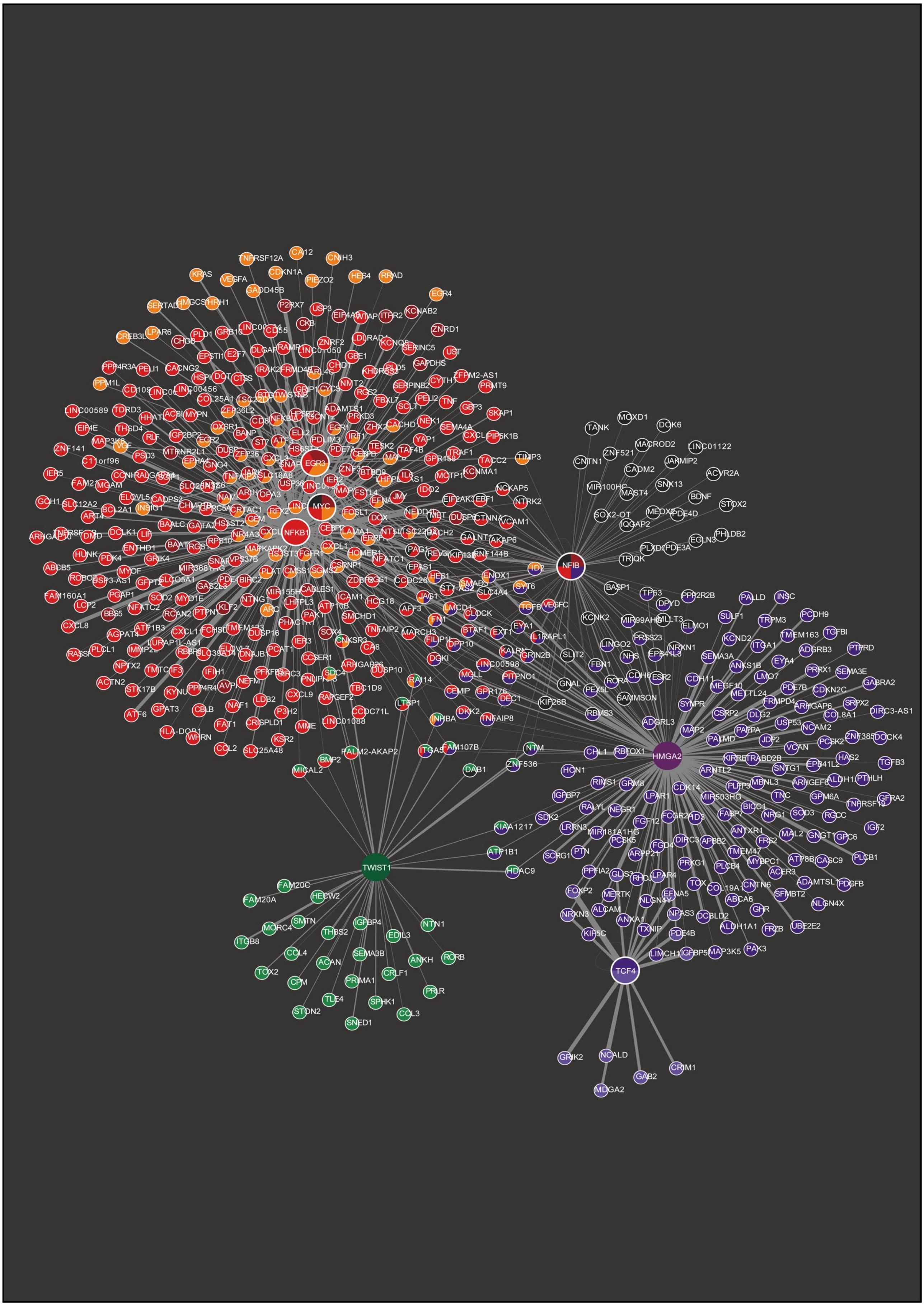
NFKB1
MYC
EGR3
NFIB
HMGA2
TWIST1
TCF4

Supplementary Figure 6. Transcription factor regulatory network analysis of plantar melanoma subgroups

pySCENIC was used to analyze transcription factor regulatory networks in melanoma subgroups involving *MYC*, *NFKB1*, *NFIB*, *EGR3*, *HMGA2*, *TCF4*, and *TWIST*. Different colors represent different gene networks regulated by specific transcription factors, with *MYC*, *NFKB1*, *NFIB*, *EGR3*, *HMGA2*, *TCF4*, and *TWIST* in dark red, red, black, ochre, purple, light purple, and green, respectively. In cases where multiple transcription factors regulate a gene, a pie chart represents the proportion of each contributing factor's color.
